# Characterization of a wheat–tetraploid *Thinopyrum elongatum* 1E(1D) substitution line K17–841-1 by cytological and phenotypic analysis and developed molecular markers

**DOI:** 10.1186/s12864-019-6359-9

**Published:** 2019-12-10

**Authors:** Daiyan Li, Juwei Zhang, Haijiao Liu, Binwen Tan, Wei Zhu, Lili Xu, Yi Wang, Jian Zeng, Xing Fan, Lina Sha, Haiqin Zhang, Jian Ma, Guoyue Chen, Yonghong Zhou, Houyang Kang

**Affiliations:** 1 State Key Laboratory of Crop Gene Exploration and Utilization in Southwest China, Sichuan Agricultural University, Chengdu, 611130 Sichuan China; 2 Triticeae Research Institute, Sichuan Agricultural University, Chengdu, 611130 Sichuan China; 3 College of Resources, Sichuan Agricultural University, Chengdu, 611130 Sichuan China

**Keywords:** Tetraploid *Thinopyrum elongatum*, Chromosome substitution line, Stripe rust, Species- specific molecular markers, GBS

## Abstract

**Background:**

Tetraploid *Thinopyrum elongatum* (2*n* = 4*x* = 28) is a promising source of useful genes, including those related to adaptability and resistance to diverse biotic (Fusarium head blight, rust, powdery mildew, and yellow dwarf virus) and abiotic (cold, drought, and salt) stresses. However, gene transfer rates are low for this species and relatively few species-specific molecular markers are available.

**Results:**

The wheat-tetraploid *Th. elongatum* line K17–841-1 derived from a cross between a hexaploid *Trititrigia* and Sichuan wheat cultivars was characterized based on sequential genomic and fluorescence in situ hybridizations and simple sequence repeat markers. We revealed that K17–841-1 is a 1E (1D) chromosomal substitution line that is highly resistant to stripe rust pathogen strains prevalent in China. By comparing the sequences generated during genotyping-by-sequencing (GBS), we obtained 597 specific fragments on the 1E chromosome of tetraploid *Th. elongatum*. A total of 235 primers were designed and 165 new *Th. elongatum*-specific markers were developed, with an efficiency of up to 70%. Marker validation analyses indicated that 25 specific markers can discriminate between the tetraploid *Th. elongatum* chromosomes and the chromosomes of other wheat-related species. An evaluation of the utility of these markers in a F_2_ breeding population suggested these markers are linked to the stripe rust resistance gene on chromosome 1E. Furthermore, 28 markers are unique to diploid *Th. elongatum*, tetraploid *Th. elongatum*, or decaploid *Thinopyrum ponticum*, which carry the E genome. Finally, 48 and 74 markers revealed polymorphisms between *Thinopyrum* E-genome- containing species and *Thinopyrum bessarabicum* (E^b^) and *Pseudoroegneria libanotica* (St), respectively.

**Conclusions:**

This new substitution line provide appropriate bridge–breeding–materials for alien gene introgression to improve wheat stripe rust resistance. The markers developed using GBS technology in this study may be useful for the high-throughput and accurate detection of tetraploid *Th. elongatum* DNA in diverse materials. They may also be relevant for investigating the genetic differences and phylogenetic relationships among E, E^b^, St, and other closely-related genomes and for further characterizing these complex species.

## Background

Common wheat (*Triticum aestivum* L., 2*n* = 6*x* = 42, AABBDD) is a staple cereal cultivated worldwide, with a predicted global grain yield of 757.4 million tons in 2019 [1]. However, the domestication of wheat decreased its genetic diversity as well as tolerance to biotic and abiotic stresses, which has restricted further improvements to wheat productivity and quality [2]. Stripe rust caused by *Puccinia striiformis* f. sp. *tritici* (*Pst*) is a serious wheat disease that threatens global wheat production [3, 4]. The identification and application of new disease-resistance genes and the development of disease-resistant cultivars represent the most effective means of decreasing the reliance on fungicides to control stripe rust in large-scale commercial production systems [5]. Wild relatives are a largely unexploited source of genes for agronomically important traits that can be transferred to common wheat via wide hybridizations to enrich wheat genetic diversity [6, 7].

*Thinopyrum elongatum* (syn. *Agropyron elongatum* or *Lophopyrum elongatum*) is a distant wild relative of common wheat and has long been the focus of wheat breeders. The taxon comprises the following three ploidy levels involving the E-genome: diploid (2*n* = 2*x* = 14, EE), tetraploid (2*n* = 4*x* = 28, EEEE), and decaploid (2*n* = 10*x* = 70, EEEEEEStStStSt) [8]. This species possesses many desirable traits, including strong adaptability, high tolerance to cold, drought, and salt stresses, and resistance to Fusarium head blight, rust, powdery mildew, and the yellow dwarf virus [9, 10]. To transfer desirable traits from *Th. elongatum* into wheat, wide hybridizations between *Th. elongatum* and common wheat began in the 1980s [8]. Progeny lines harboring *Th. elongatum* chromosomes (segments) incorporated into the wheat genome were obtained as lines with chromosomal additions, substitutions, or translocations [7, 11–14]. However, these introgressions mainly involved the diploid *Th. elongatum* and decaploid *Thinopyrum ponticum*. There are relatively few reports describing attempts to transfer tetraploid *Th. elongatum* genes into wheat [15–18]. Thus, identifying new elite alien genes and incorporating them into common wheat are critical for increasing wheat genetic diversity through the development of wheat–tetraploid *Th. elongatum* introgression lines.

Marker-assisted selection is useful for detecting genes associated with a trait of interest based on a linked marker, with implications for breeding involving multiple traits [11]. Developing species- specific molecular markers that facilitate the identification of alien chromosomes or segments is very important for wheat breeding programs [19, 20]. Diverse molecular markers specific to diploid *Th. elongatum* and *Th. ponticum* have been reported, including random-amplified polymorphic DNAs (RAPDs), simple sequence repeats (SSRs), expressed sequence tags, cleaved amplified polymorphic sequences, sequence-tagged sites, sequence-characterized amplified regions, amplified fragment length polymorphisms, single nucleotide polymorphisms, and specific-locus amplified fragments (SLAFs) [11, 12, 19, 21–25]. However, there are still relatively few of these markers, and they do not cover the whole *Thinopyrum* genome. Consequently, there is an urgent need to develop more molecular markers, especially those with a wide genomic distribution and that are amenable to high-throughput genotyping [12]. Genotyping-by-sequencing (GBS) is a high-throughput, highly accurate, inexpensive, and relatively simple method for developing several markers in Triticale species [26]. However, developing specific molecular markers for tetraploid *Th. elongatum* remains difficult. Therefore, generating chromosome-specific molecular markers is critical for detecting and tracing alien chromosomes in wheat–tetraploid *Th. elongatum* hybrid derivatives.

Tetraploid *Th. elongatum* is an important genetic resource for improving wheat, and some wheat–tetraploid *Th. elongatum* derivative lines have been developed by crossing hexaploid *Trititrigia* with Sichuan wheat cultivars [17]. Li et al. [18] produced a fluorescence in situ hybridization (FISH) karyotype of the E-genome chromosomes of tetraploid *Th. elongatum* based on various repetitive sequence probes, which may enhance the utility of tetraploid *Th. elongatum* for the introgression of alien genes into wheat. The main objectives of the current study were to: (1) characterize the chromosomal constitution of a wheat–tetraploid *Th. elongatum* substitution line and evaluate its effects on stripe rust resistance and agronomic traits and (2) develop and validate specific and easy-to-use molecular markers based on GBS, which may be useful for the efficient and reliable identification of tetraploid *Th. elongatum* chromatin in several species, including common wheat.

## Results

### Chromosomal constitution of K17–841-1

**Fig. 1:**
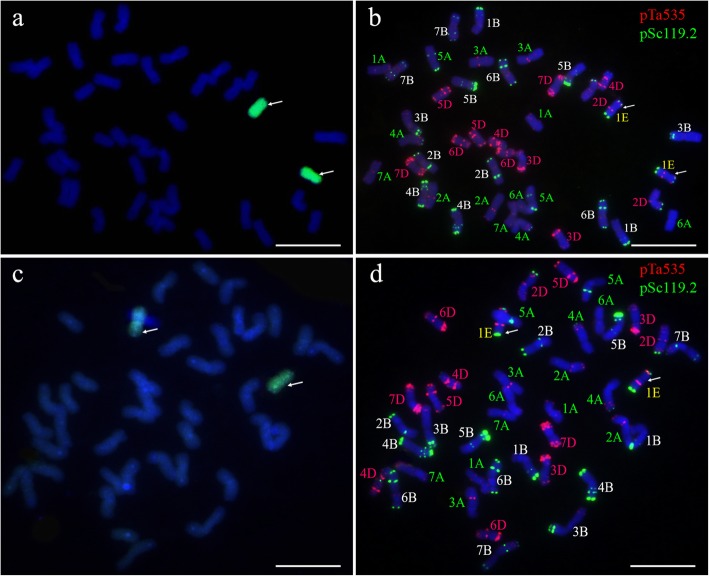
GISH and FISH identification of the wheat–tetraploid *Th. elongatum* substitution line K17–841-1. The probes used for in situ hybridization were tetraploid *Th. elongatum* genomic DNA (**a**, **c)**; pSc119.2 and pTa535 (**b**, **d**). Arrows indicate 1E-genome chromosomes. Scale bar: 10 μm

Genomic in situ hybridization (GISH), FISH, and SSR marker analyses were performed to determine the chromosomal constitution of wheat–tetraploid *Th. elongatum* line K17–841-1. When tetraploid *Th. elongatum* total genomic DNA and the J-11 genomic DNA were used as the probe and the blocking DNA, respectively, we observed that line K17–841-1 carried 40 wheat chromosomes and two E chromosomes (Fig. 1a). The GISH signals were sequentially removed and the slides were used in a FISH analysis involving pSc119.2 and pTa535 probes. A pair of E chromosomes produced strong terminal pSc119.2 signals on both arms as well as strong pTa535 signals on the subterminal regions of the short arm and near the centromeric region of the long arm (Fig. 1b). These results are consistent with the previously reported FISH pattern for the 1E chromosome of tetraploid *Th. elongatum* [18]. Thus, the FISH karyotype of the E-genome chromosomes of tetraploid *Th. elongatum* and common wheat based on probes pSc119.2 and pTa535 suggested that K17–841-1 is a 1E (1D) chromosomal substitution line. To determine the cytological stability of K17–841-1, we used GISH and FISH to analyze 20 randomly selected seeds of K17–841-1 self-progeny. We revealed that all seeds contained 14 A- (1A–7A), 14 B- (1B–7B), and 12 D- (2D–7D) genomes, as well as a pair of 1E chromosomes (Fig. 1c, d).

**Fig. 2:**
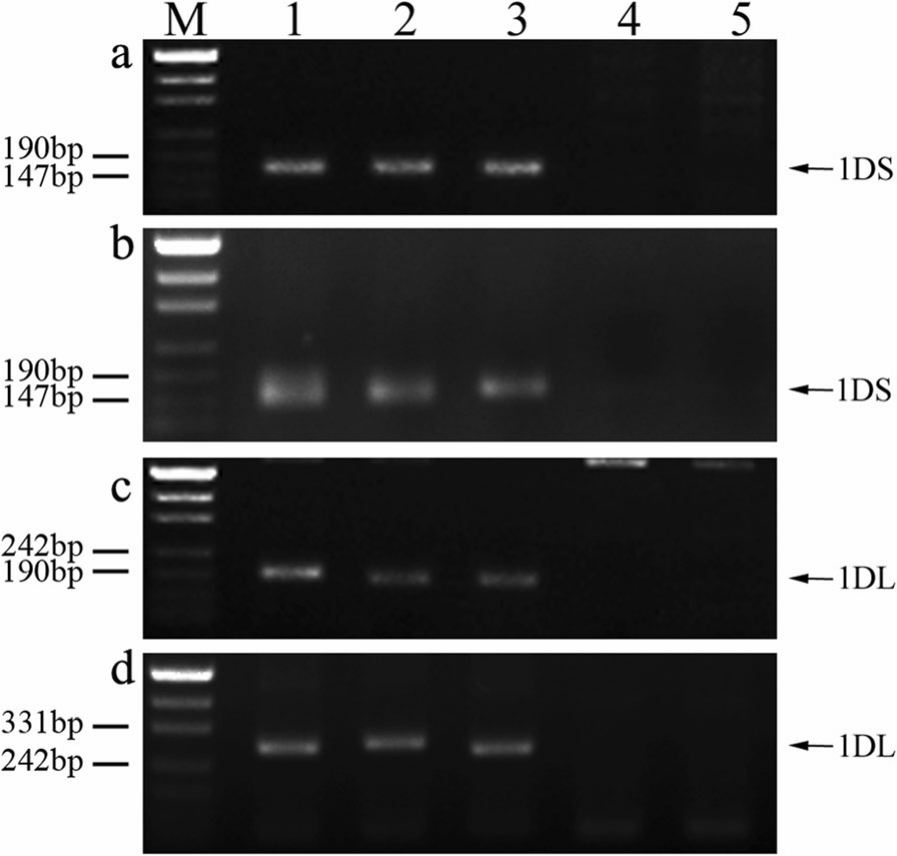
PCR amplification of SSR markers *wmc147* (**a**), wmc222 (**b**), *gwm337* (**c**), and *Xcfd63* (**d**). *Lanes M* marker, *1* CS, *2* wheat cultivar Shumai482, *3* wheat cultivar Shumai921, *4 *8801 (*T. durum*-tetraploid *Th. elongatum* amphidiploid), *5* K17–841-1(wheat–tetraploid *Th. elongatum* substitution line). Arrows show the diagnostic amplification products of SSR markers

We completed a PCR analysis involving the wheat chromosome 1D-specific SSR markers to confirm the chromosomal constitution of K17–841-1. As expected, amplified products for the chromosome 1D SSR markers (i.e., *wmc147*, *wmc222*, *gwm337*, and *Xcfd63*) were detected for Chinese Spring (CS), Shumai482 (SM482), and Shumai921 (SM921). In contrast, amplicons were not genetated for 8801 and K17–841-1 (Fig. 2). Our results verified that in line K17–841-1, wheat chromosome 1D had been replaced by chromosome 1E of tetraploid *Th. elongatum*.

### Morphology of K17–841-1

**Fig. 3:**
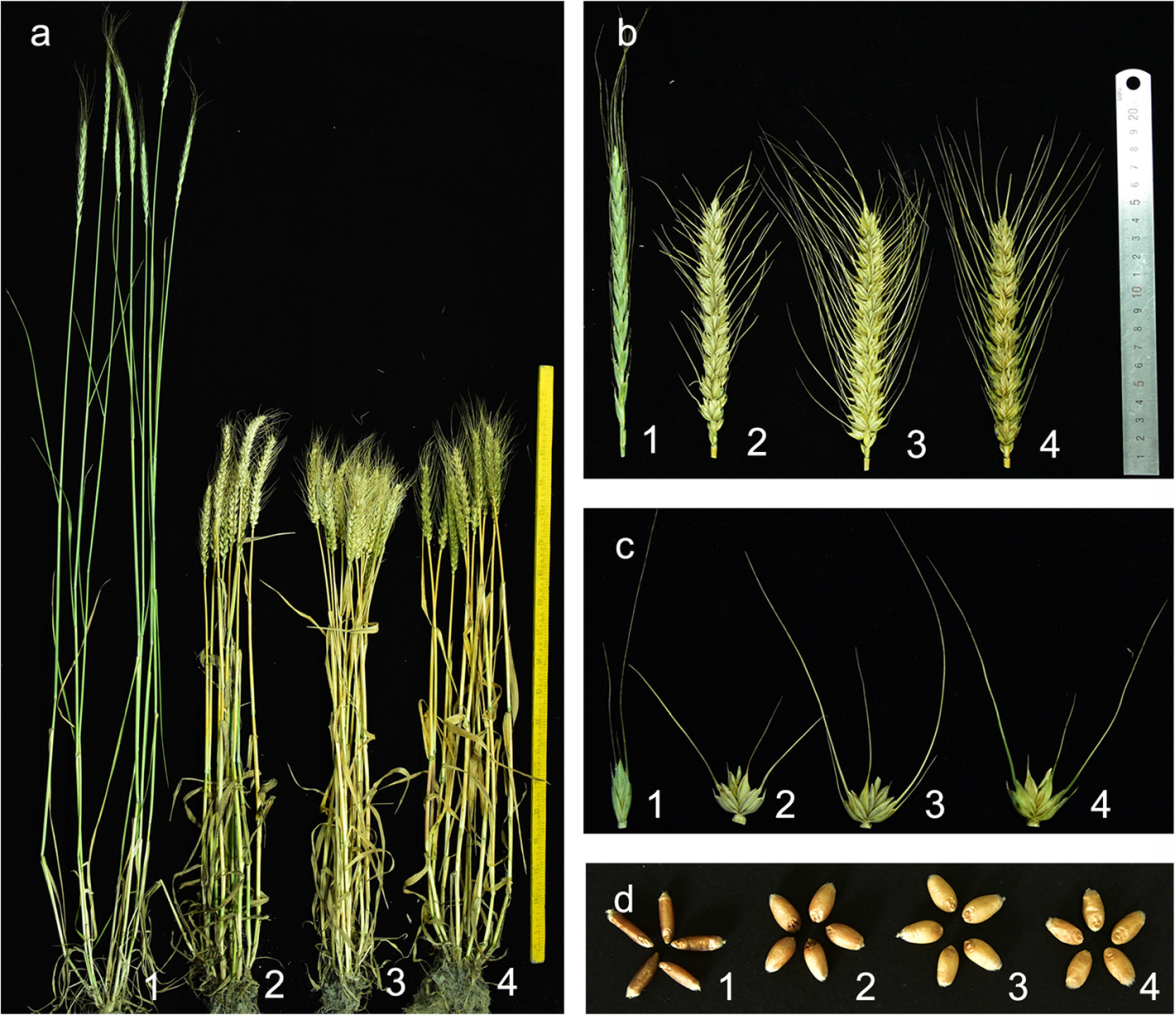
Plant morphology of the wheat–tetraploid *Th. elongatum* substitution line K17–841-1 and its parents. Adult plants (**a**), spikes (**b**), spikelets(**c**), and grains(**d**). Numbers 1–4 represent 8801 (*T. durum*-tetraploid *Th. elongatum* amphidiploid), wheat cultivar Shumai482, wheat cultivar Shumai 921, and K17–841-1, respectively

We analyzed the agronomic traits of K17–841-1 and the donor parents in two growing seasons. Line K17–841-1 displayed stable morphological traits, which were similar to those of the wheat parents SM482 and SM921 (Table 1**;** Fig. 3). The average plant height and spike length of line K17–841-1 were significantly lower than those of the *Triticum durum*–tetraploid *Th. elongatum* partial amphidiploid 8801, but were greater than those of SM482 and similar to those of SM921 (Fig. 3a, b). The number of spikelets per spike was lower than that of SM482 or SM921, but was similar to that of 8801. The grain number per spike was greater than that of 8801, but was lower than that of SM921 and similar to that of SM482 (Fig. 3c). There were no significant differences between K17–841-1 and either SM482 or SM921 regarding the tiller number, thousand-kernel weight, and seed setting rate (Fig. 3d).

**Table 1:** Agronomic traits of K17–841-1 and its parental lines

Lines	Year	Plant height (cm)	Tiller number	Spike length (cm)	Spikelet per spike	Grains per spike	Thousand-grain weight (g)	Seed setting rate (%)
8801	2017–2018	143.6 ± 6.0a	8.2 ± 2.3a	15.5 ± 2.1a	17.5 ± 1.2c	39.7 ± 2.9c	24.5 ± 0.5b	84.0 ± 8.4b
	2018–2019	132.4 ± 4.5a	8.5 ± 0.6a	17.3 ± 2.1a	17.5 ± 1.3b	31.3 ± 5.0c	29.3 ± 0.2b	68.5 ± 12.8b
SM482	2017–2018	82.5 ± 4.3c	9.2 ± 1.6a	13.4 ± 1.3b	22.0 ± 2.1b	63.3 ± 9.4b	44.7 ± 0.2a	99.6 ± 0.9a
	2018–2019	79.0 ± 5.2c	8.8 ± 0.4a	12.5 ± 0.9b	18.6 ± 1.7ab	58.2 ± 7.9b	43.2 ± 0.8a	97.9 ± 2.3a
SM921	2017–2018	86.1 ± 3.1bc	9.3 ± 2.3a	13.9 ± 1.4ab	24.7 ± 2.9a	84.3 ± 12.3a	44.5 ± 2.0a	99.4 ± 1.6a
	2018–2019	87.7 ± 2.6b	4.8 ± 1.3b	13.2 ± 0.8b	20.6 ± 0.9a	72.8 ± 8.6a	38.6 ± 0.8b	92.7 ± 3.2a
K17–841-1	2017–2018	89.9 ± 4.2b	8.2 ± 1.3a	13.5 ± 0.8b	19.5 ± 1.0c	63.0 ± 8.2b	44.6 ± 1.7a	95.7 ± 1.3a
	2018–2019	94.1 ± 4.8b	9.3 ± 1.8a	14.0 ± 0.6b	17.8 ± 1.0b	51.8 ± 11.0b	44.3 ± 1.2a	93.8 ± 5.4a

Data in the columns indicate means ± standard errors

Lowercase letters following the means indicate significant differences at *P* < 0.05 as determined by the least significant differences

### Stripe rust resistance evaluation

**Fig. 4:**
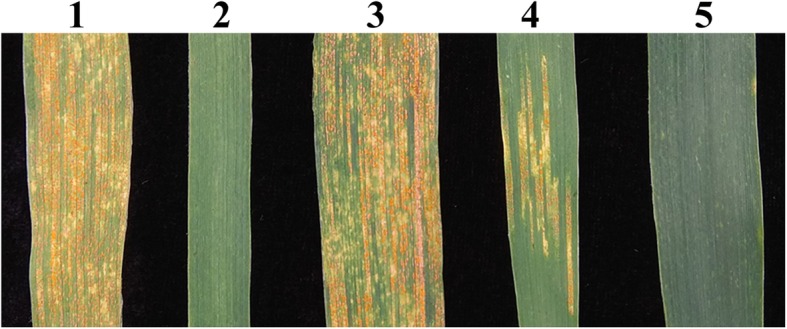
Stripe rust resistance of the wheat–tetraploid *Th. elongatum* substitution line K17–841-1 and the controls. *1* wheat line SY95–71, *2 *8801 (*T. durum*-tetraploid *Th. elongatum* amphidiploid), *3* wheat cultivar Shumai482, *4* wheat cultivar Shumai921, *5* K17–841-1

We evaluated the stripe rust resistance of K17–841-1, 8801, SM482, SM921, and SY95–71 plants inoculated with a mixture of *Pst* races (CYR-32, CYR-33, CYR-34, and V26/Gui22–14) at Chengdu, Sichuan, China. An analysis of three replicates revealed that the adult SM482, SM921, and SY95–71 plants were susceptible to these *Pst* races, with infection types (ITs) of 4, 3, and 4, respectively. In contrast, 8801 and K17–841-1 plants were highly resistant to these races (i.e., IT of 0) (Fig. 4).

### Development and validation of specific molecular markers for the 1E chromosome of tetraploid *Th. elongatum*

The GBS approach was applied to identify tetraploid *Th. elongatum* 1E chromosome-specific sequences. Details regarding the sequencing, including raw reads, clean reads, effective rate, error rate, Q20, Q30, and GC content, are summarized in Table 2. The results revealed a high sequencing quality (Q20 ≥ 94% and Q30 ≥ 86%) and a normal GC content distribution. A total of 45,115,502, 7,389,702, 8,710,288, and 8,831,260 effective GBS sequences were obtained for PI531718, PI531750, 8801, and K17–841-1, respectively (Table 3). The sequencing depth was more than 10.32×. A sequence comparison revealed 73,744 K17–841-1 clean reads that were less than 23% homologous with CS sequences. Additional sequence alignments uncovered 2952 K17–841-1 reads that were more than 23% homologous with 8801 and PI531750 fragments obtained by GBS. Finally, 1194 K17–841-1 unique reads (597 specific fragments) that were less than 10% homologous with sequences from PI531718 and the other six substitution lines were obtained and were considered the tetraploid *Th. elongatum* 1E chromosome-specific fragments.

**Table 2:** Quality of GBS data

Genotype	Raw base (bp)	Clean base (bp)	Effective rate (%)	Error rate (%)	Q20 (%)	Q30 (%)	GC (%)
PI531718	6,496,632,288	6,496,632,288	100	0.03	95.97	93.68	43.95
PI531750	1,064,120,832	1,064,117,088	100	0.04	95.96	89.20	42.99
8801	1,254,284,064	1,254,281,472	100	0.05	95.00	87.17	42.13
K17–841-1	1,271,704,608	1,271,701,440	100	0.05	94.71	86.58	43.17

**Table 3:** Sequence alignment between K17–841-1 and its parental lines

Genotype	Total reads	Unmapped reads	Map2 parent reads	uniqReads
PI531718	45,115,502	5,309,332		
PI531750	7,389,702	776,849		
8801	8,710,288	300,048		
K17–841-1	8,831,260	73,744	2952	1194

Unmapped reads: reads unmapped on CS

Map 2 parent reads: reads mapped on PI531750 and 8801

UniqReads: unique reads of K17–841-1

**Fig. 5:**
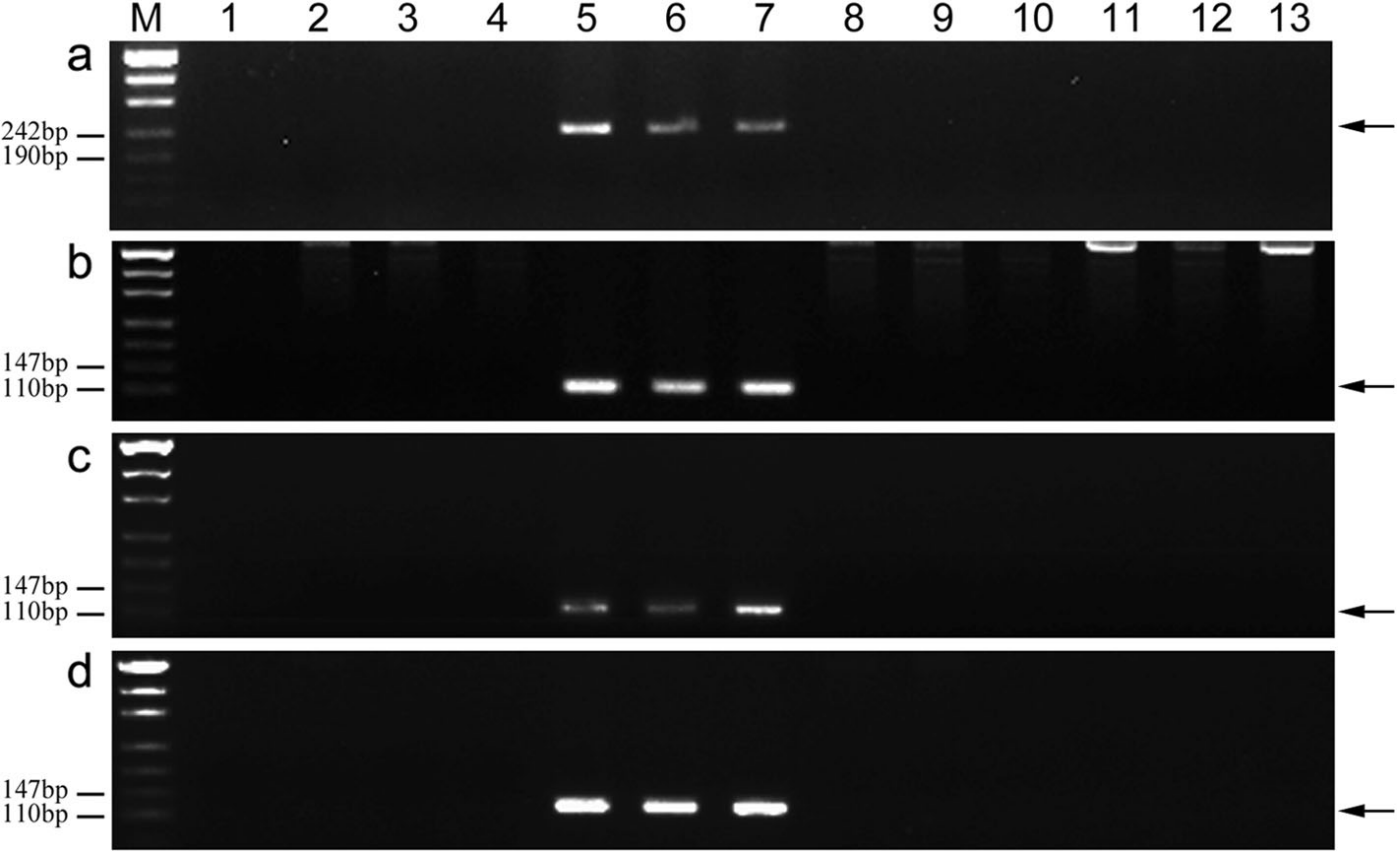
PCR amplification of markers TTE1E-12 (**a**), TTE1E-140 (**b**), TTE1E-189 (**c**), and TTE1E-193 (**d)**. *Lanes M* marker, *1* CS, *2* wheat cultivar Shumai482, *3* wheat cultivar Shumai921, *4* diploid *Thinopyrum elongatum*, *5* tetraploid *Thinopyrum elongatum*, *6 *8801 (*T. durum*-tetraploid *Th. elongatum* amphidiploid), *7* K17–841-1 (wheat–tetraploid *Th. elongatum* substitution line), *8* TDS2E(2A), *9* TDS3E(3D), *10* TDS4E(4D), *11* TDS5E(5D), *12* TDS6E(6D), *13* TDS7E(7D). Arrows show the diagnostic amplification products of tetraploid *Th. elongatum* 1E chromosome

**Fig. 6:**
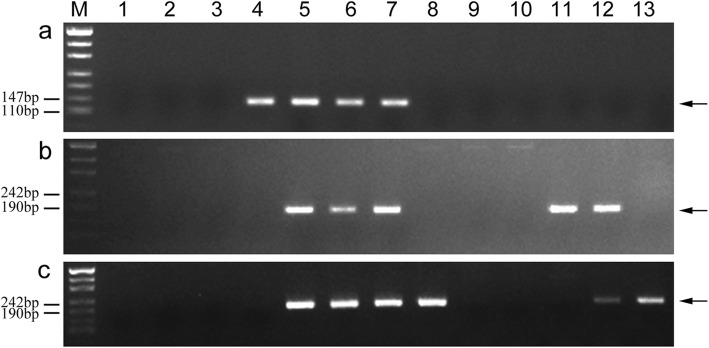
PCR amplification of markers TTE1E-214 (**a**), TTE1E-3 (**b**), and TTE1E-58 (**c**). *Lanes M* marker, *1* CS, *2* wheat cultivar Shumai482, *3* wheat cultivar Shumai921, *4* diploid *Thinopyrum elongatum*, *5* tetraploid *Thinopyrum elongatum*, *6 *8801 (*T. durum*-tetraploid *Th. elongatum* amphidiploid), *7* K17–841-1 (wheat–tetraploid *Th. elongatum* substitution line), *8* TDS2E(2A), *9* TDS3E(3D), *10* TDS4E(4D), *11* TDS5E(5D), *12* TDS6E(6D), *13* TDS7E(7D). Arrows show the diagnostic amplification products of tetraploid *Th. elongatum* 1E chromosome

To develop tetraploid *Th. elongatum* 1E chromosome-specific markers, 235 PCR primers pairs were designed based on these specific fragments and used to amplify sequences from CS, SM482, SM921, PI531718, PI531750, 8801, K17–841-1, and six wheat-tetraploid *Th. elongatum* disomic substitution (TDS) lines (2E-7E). A total of 165 (70%) *Th. elongatum-*specific molecular markers were successfully developed (see Additional file 1: Table S1), of which 132 markers amplified specific sequences only from PI531750, 8801, and K17–841-1 (Type I) (Fig. 5a-d). Therefore, these markers were regarded as tetraploid *Th. elongatum* 1E chromosome-specific molecular markers, with a success rate of up to 56.2%. Additionally, 21 markers amplified specific sequences only from PI531718, PI531750, 8801, and K17–841-1 (Type II) (Fig. 6a). Moreover, 12 markers were also detected on the other E chromosomes of tetraploid *Th. elongatum* (Fig. 6b, c).

**Fig. 7:**
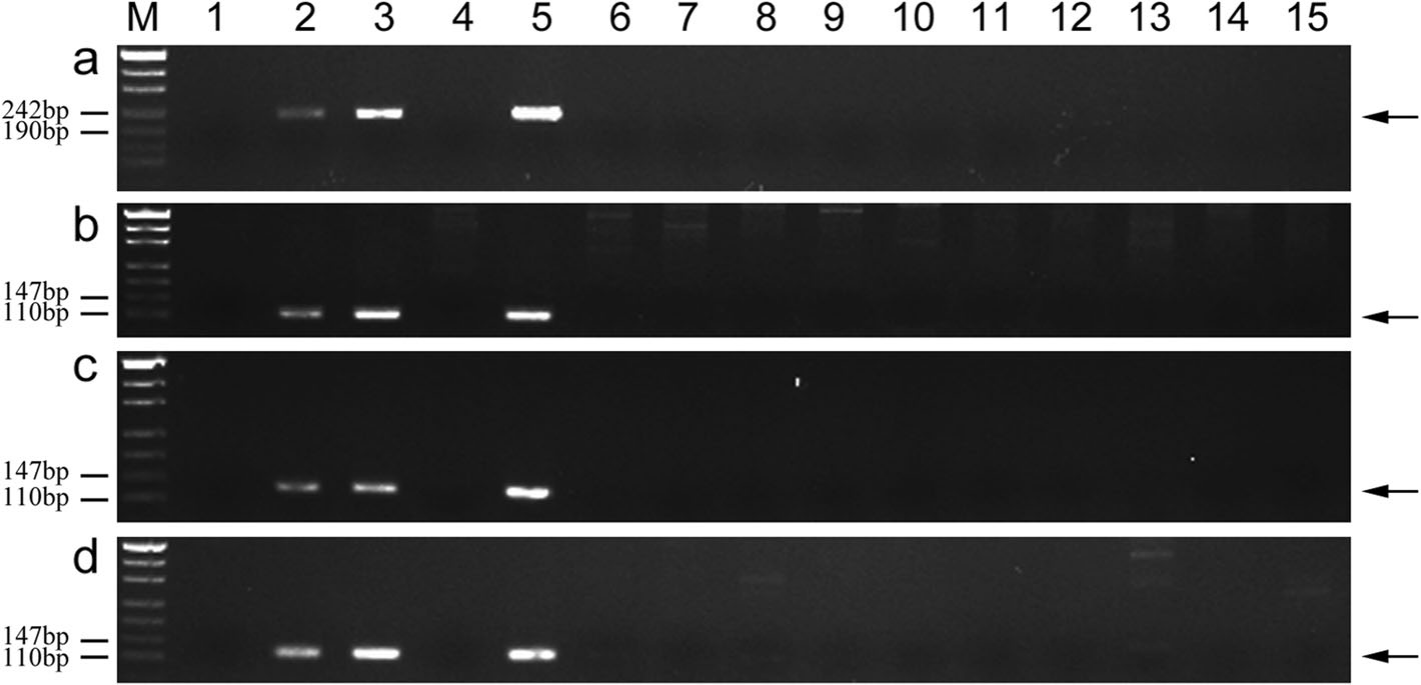
Stability and specificity of markers TTE1E-12 (**a**), TTE1E-140 (**b**), TTE1E-189 (**c**), and TTE1E-193 (**d)** in other wheat-related species. *Lanes M* marker, *1* CS, *2 *8801 (*T. durum*-tetraploid *Th. elongatum* amphidiploid), *3* K17–841-1 (wheat–tetraploid *Th. elongatum* substitution line), *4* diploid *Thinopyrum elongatum*, *5* tetraploid *Thinopyrum elongatum*, *6 Thinopyrum ponticum*, *7 Thinopyrum bessarabicum*, *8 Pseudoroegneria libanotica*, *9 Dasypyrum villosum*, *10 Hordeum bogdanii*, *11 Agropyron cristatum*, *12 Secale cereale*, *13 Psathyrostachys huashanica*, *14 Trichopyrum caespitosum*, *15 Psammopyrum athericum*. Arrows show the diagnostic amplification products of tetraploid *Th. elongatum* 1E chromosome

**Fig. 8:**
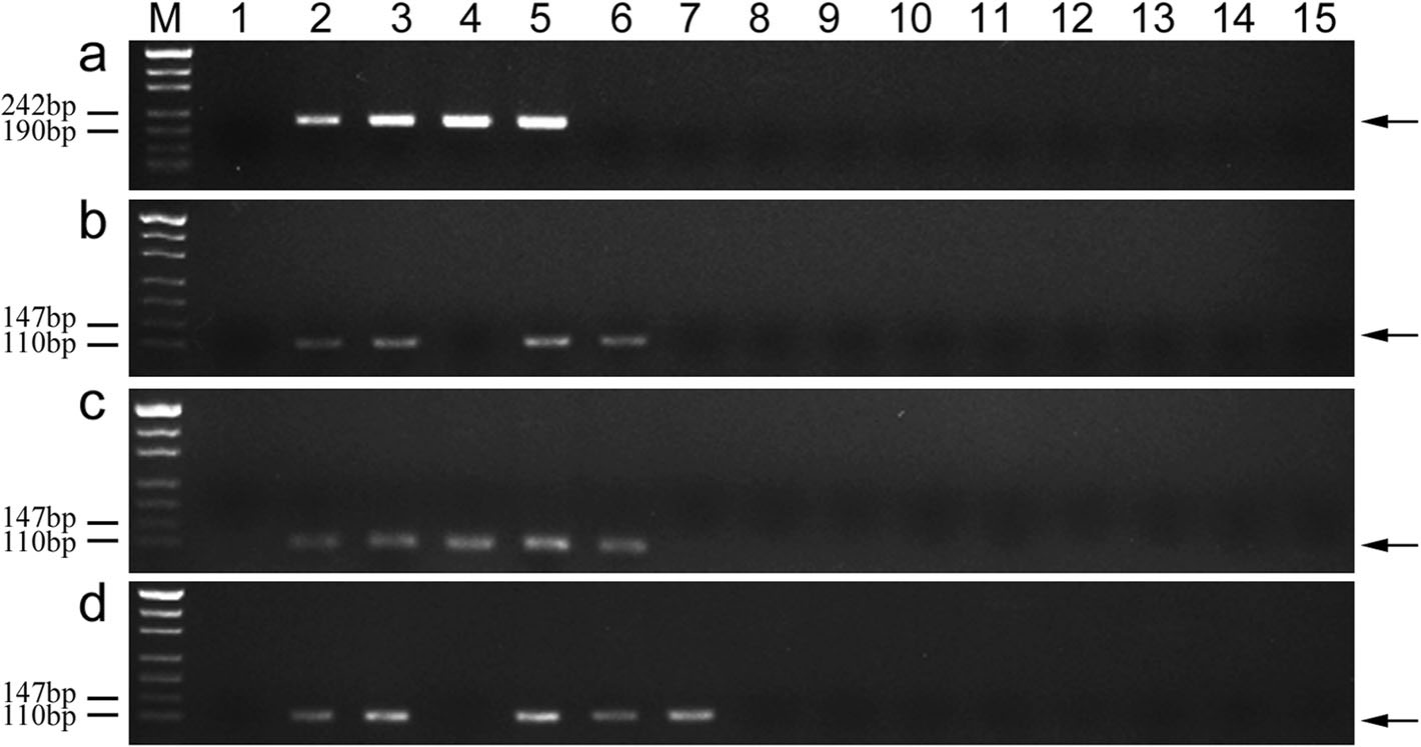
Stability and specificity of markers TTE1E-59 (**a**), TTE1E-184 (**b**), TTE1E-222 (**c**), and TTE1E-192 (**d**) in other wheat-related species. *Lanes M* marker, *1* CS, *2 *8801 (*T. durum*-tetraploid *Th. elongatum* amphidiploid), *3* K17–841-1 (wheat–tetraploid *Th. elongatum* substitution line), *4* diploid *Thinopyrum elongatum*, *5* tetraploid *Thinopyrum elongatum*, *6 Thinopyrum ponticum*, *7 Thinopyrum bessarabicum*, *8 Pseudoroegneria libanotica*, *9 Dasypyrum villosum*, *10 Hordeum bogdanii*, *11 Agropyron cristatum*, *12 Secale cereale*, *13 Psathyrostachys huashanica*, *14 Trichopyrum caespitosum*, *15 Psammopyrum athericum*. Arrows show the diagnostic amplification products of tetraploid *Th. elongatum* 1E chromosome

To confirm marker specificity and stability, 153 markers (Types I and II) were used to further analyze 11 wheat-related species. The PCR results are presented in Additional file 2: Table S2. Among the 132 tetraploid *Th. elongatum* 1E chromosome-specific markers, 25 amplified sequences from tetraploid *Th. elongatum*, but not from the analyzed related species (Table 4; Fig. 7a-d). In contrast, 21 and 106 markers amplified specific sequences from not only tetraploid *Th. elongatum*, but also from diploid *Th. elongatum* and decaploid *Th. ponticum*, respectively (Fig. 8a, b). Additionally, 28 markers amplified a common sequence from tetraploid *Th. elongatum*, diploid *Th. elongatum*, and *Th. ponticum*, but did not amplify any sequences from the other related species (Fig. 8c). Five markers amplified specific sequences not only from tetraploid *Th. elongatum* and *Th. ponticum*, but also from *Thinopyrum bessarabicum* (Fig. 8d). Four markers amplified a common sequence from diploid *Th. elongatum*, tetraploid *Th. elongatum*, *Th. ponticum*, *Th. bessarabicum*, *Pseudoroegneria libanotica*, *Trichopyrum caespitosum*, and *Psammopyrum athericum*. Furthermore, 58, 32, 5, 8, 28, 8, 13, 50, and 73 markers amplified sequences from *Th. bessarabicum*, *Pse. libanotica*, *Dasypyrum villosum*, *Hordeum bogdanii*, *Agropyron cristatum*, *Secale cereale*, *Psathyrostachys huashanica*, *Tr. caespitosum*, and *Psa. athericum*, respectively.

**Table 4:** Specific amplification results of 1E-chromosomal markers in wheat-related species

Number of markers	Amplification result											
	PI531718 (E)	PI531750 (EE)	PI531737 (EEEEE)	W6–10232 (E^b^)	PI228391 (St)	PI251477 (V)	Y1819 (H)	PI610892 (P)	QL (R)	ZY3156 (Ns)	PI634264 (StE)	PI531744 (StEP)
25	0	1	0	0	0	0	0	0	0	0	0	0
2	1	1	0	0	0	0	0	0	0	0	0	0
24	0	1	1	0	0	0	0	0	0	0	0	0
2	1	1	1	0	0	0	0	0	0	0	0	0
0	1	1	1	1	0	0	0	0	0	0	0	0
5	0	1	1	1	0	0	0	0	0	0	0	0
2	0	1	1	1	1	0	0	0	0	0	1	1
4	1	1	1	1	1	0	0	0	0	0	1	1
2	1	1	0	0	1	0	1	1	0	0	0	0
5	0	1	1	0	0	0	0	0	0	0	1	1
11	0	1	1	1	0	0	0	0	0	0	0	1
4	0	1	1	1	0	0	0	0	0	0	1	1
1	0	1	0	0	0	0	0	0	0	1	0	0

“1” or “0” indicates the presence or absence of the specific band, respectively

### Utility of the tetraploid *Th. elongatum*-specific markers in the F_2_ population

**Fig. 9:**
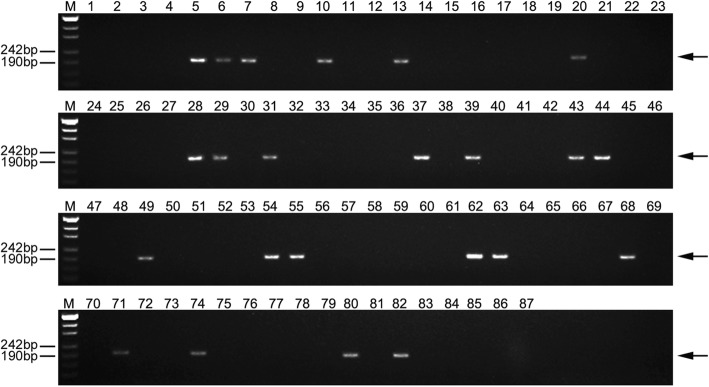
Utility of marker TTE1E-21 in 80 F_2_ individuals of K17–841-1 and SM969. *1* CS, *2* wheat cultivar Shumai482, *3* wheat cultivar Shumai921, *4* wheat cultivar Shumai969, *5* tetraploid *Thinopyrum elongatum*, *6 *8801 (*T. durum*-tetraploid *Th. elongatum* amphidiploid), *7* K17–841-1 (wheat–tetraploid *Th. elongatum* substitution line), *8–87* 80 F_2_ individuals. Arrows show the diagnostic amplification products of tetraploid *Th. elongatum* 1E chromosome

**Fig. 10:**
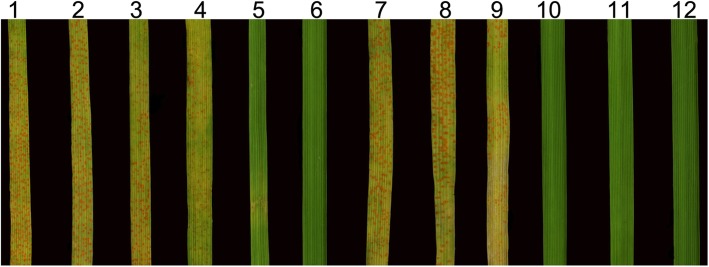
Evaluation for seedling stage reacctions to *Pst* race CYR-34 in F_2_ individuals of K17–841-1 and SM969 and the controls. *1* wheat line SY95–71, *2* wheat cultivar Shumai482, *3* wheat cultivar Shumai921, *4* wheat cultivar Shumai969, *5 *8801 (*T. durum*-tetraploid *Th. elongatum* amphidiploid), *6* K17–841-1 (wheat–tetraploid *Th. elongatum* substitution line), *7–9* susceptible F_2_ individuals, *10–12* resistant F_2_ individuals

To verify the utility of the newly developed molecular markers, 80 F_2_ individuals of K17–841-1 and wheat cultivar Shumai969 (SM969) were analyzed by PCR. We detected specific amplicons for only 20 individuals (Fig. 9, Additional file 3: Table S3). The results of a GISH indicated these 20 plants with specific amplicons carried 1E (1D) chromosomal substitutions, while the 60 plants without specific amplicons had no GISH signal in their chromosome preparation. More importantly, an evaluation of stripe rust resistance at the seedling stage revealed that 8801, K17–841-1, and the 20 positive F_2_ individuals carrying 1E chromosomal markers were highly resistant to *Pst* race CYR-34. In contrast, the 60 F_2_ plants lacking amplicons as well as SY95–71 and the parental lines SM482, SM921, and SM969 were highly susceptible (Fig. 10, Additional file 3: Table S3). These observations implied that the specific markers developed in this study may be useful for detecting the stripe rust resistance gene on the 1E chromosome of tetraploid *Th. elongatum* during the breeding of new disease-resistant wheat varieties.

## Discussion

Distant hybridizations may be useful for transferring agronomically valuable genes from wild relatives to common wheat varieties. Creating intermediate lines carrying alien chromosomes of wheat relatives provides the basis for using these germplasm resources to improve domesticated wheat [27]. Wheat–alien chromosomal substitution lines are important bridge materials for transferring alien genes to common wheat. In recent decades, there have been many attempts by wheat breeders to generate wheat–*Th. elongatum* substitution lines [8]. However, most of the introgressions from *Th. elongatum* into wheat involved the diploid *Th. elongatum* and decaploid *Th. ponticum*. For example, CS–diploid *Th. elongatum* addition and substitution lines have been produced [28]. Additionally, Zheng et al. [29] generated the wheat–*Th. ponticum* 4Ag (4D) disomic substitution line Blue 58 from a hybridization between *Th. ponticum* and common wheat. Fu et al. [30] developed a wheat–diploid *Th. elongatum* 7E (7D) substitution line resistant to Fusarium head blight. Wang et al. [31] produced a wheat–*Th. ponticum* St (6A) disomic substitution line exhibiting powdery mildew resistance. The 7J^St^ (7B) substitution line CH1113-B13, which is resistant to stripe rust, was identified from among the progeny of a cross between common wheat and *Th. ponticum* [32]. The tetraploid *Th. elongatum* harbors many beneficial genes that provide protection from diseases as well as cold, drought, and salinity stresses. Thus, it is a valuable genetic resource for improving the tolerance of common wheat to biotic and abiotic stresses [9, 10, 17]. To date, there are few reports regarding wheat–tetraploid *Th. elongatum* substitution lines. Li et al. [17] developed 50 wheat–tetraploid *Th. elongatum* introgression lines by crossing the *T. durum*–tetraploid *Th. elongatum* partial amphidiploid line 8801 with wheat varieties native to the Sichuan Basin, China. These lines comprise 40–47 chromosomes and most of them are cytologically unstable. In the current study, we further characterized the wheat–tetraploid *Th. elongatum* 1E (1D) disomic substitution line K17–841-1 derived from the 8801/SM482/SM921 F_5_ progeny. The FISH and SSR analyses confirmed that common wheat chromosome 1D was substituted with the homologous chromosome 1E from tetraploid *Th. elongatum*, providing further evidence that the E genome of *Th. elongatum* is closely related to the D genome of common wheat [33, 34]. Interestingly, an evaluation of the stripe rust resistance indicated that the wheat parents SM482 and SM921 are susceptible and that the resistance of K17–841-1 was derived from tetraploid *Th. elongatum*. Some stripe rust resistance genes have been identified in *Thinopyrum* species and have been transferred to common wheat, including *Yr50* from *Th. intermedium*, *YrE* from diploid *Th. elongatum*, and *Yr69*, *YrTP1*, *YrTP2*, and *YrCH7056* from *Th. ponticum* [35, 36]. To the best of our knowledge, this study is the first to demonstrate the successful transfer of a new and high-level stripe rust resistance gene from tetraploid *Th. elongatum*. Furthermore, the grain yield of line K17–841-1 is similar to that of the wheat parents SM482 and SM921. Accordingly, K17–841-1 represents an appropriate bridge breeding material for the introgression of alien genes to enhance wheat disease resistance.

Molecular markers are widely used to detect and track alien chromosomes and/or chromosomal segments during crossing and selection [10]. Conventional methods for developing markers, including SSRs, RAPDs, and amplified fragment length polymorphisms, have low success rates because of the high genomic sequence homology between *Th. elongatum* and common wheat. For example, using 40 SSR primers, You et al. [37] amplified 108 diploid *Th. elongatum-*specific fragments, but only one genome-specific molecular marker was developed. In another previous study, 94 diploid *Th. elongatum*-specific fragments were generated with 26 pairs of RAPD primers, but only three 1E or 3E chromosome-specific markers were developed [38]. Additionally, Ge et al. [39] developed one diploid *Th. elongatum*-specific marker from 65 specific fragments based on suppression subtractive hybridization. Furthermore, inter-SSR, inter-retrotransposon amplified polymorphism, and retrotransposon microsatellite amplified polymorphism techniques were applied to develop 13 specific sequence-characterized amplified region markers based on 34 specific diploid *Th. elongatum* sequences [40]. In a recent study, 135 randomly selected fragments of diploid *Th. elongatum* generated by SLAF-sequencing (SLAF-seq) were used to develop 89 specific molecular markers, including 61 7E chromosome-specific markers [11]. Chen et al. [25] developed 20 diploid *Th. elongatum* 1E chromosome-specific and two E genome-specific molecular markers based on SLAF-seq data, with an efficiency of up to 60%. Approximately 89% of the mined SNPs of diploid *Th. elongatum* may be authentic with respect to their polymorphisms and chromosomal locations as determined by a high-resolution melting curve assay [12]. Moreover, SLAF-seq data were used to develop 67 *Th. ponticum*-specific molecular markers from a wheat–*Th. ponticum* translocation line, with a success rate of up to 39% [19]. Furthermore, GBS, which is a robust, simple, and high-throughput next-generation sequencing approach that can decrease the complexity of large genomes, has been applied to develop many molecular markers in Triticale species [26, 41]. This approach can generate considerable sequence information and is appropriate for all whole-genome density distributions. The flexibility and low cost of GBS makes it ideal for genomics-assisted breeding [42]. To date, there have been no reports describing the development of tetraploid *Th. elongatum*-specific molecular markers. In this study, the GBS method was applied to develop 165 specific molecular markers, including 132 tetraploid *Th. elongatum* chromosome 1E-specific PCR markers, with a success rate of up to 70%. The greater success rate for our study relative to those of earlier investigations indicates that GBS technology may be used to efficiently develop alien-specific molecular markers in a wheat background.. The markers developed in this study may be useful for the high-throughput and accurate detection of tetraploid *Th. elongatum* DNA in diverse materials. Most importantly, the verification of their utility for analyzing a F_2_ breeding population implies that these specific markers are appropriate for tracing the stripe rust resistance gene carried on chromosome 1E of tetraploid *Th. elongatum* for the breeding of enhanced materials.

Even after removing K17–841-1 sequences that were more than 90% homologous with sequences from six other substitution lines (2E–7E), 12 markers still amplified specific sequences in the other substitution lines, confirming that the *Th. elongatum* E chromosomes sequences are highly homologous, likely because of chromosomal rearrangements. Earlier investigations involving biochemical and molecular markers as well as SLAF-seq revealed similar sequence identity levels for the diploid *Th. elongatum* [11, 43]. After analyzing the hybrids from crosses between tetraploid *Th. elongatum* and diploid *Th. elongatum* and *Th. bessarabicum*, Dvořák [44] concluded that tetraploid *Th. elongatum* is a segmental allotetraploid with two closely related E genomes. On the basis of examinations of chromosomal pairs, karyotypes, and C-banding patterns,Liu and Wang [45] also determined that tetraploid *Th. elongatum* is a segmental allotetraploid carrying two E genomes with slight differences. In a previous study involving a FISH with repetitive clones, two tetraploid *Th. elongatum* accessions were analyzed to uncover the significant chromosomal polymorphisms between the E genome of the putative diploid progenitor *Th. elongatum* and the other genome [18]. The verification of 153 markers specific to tetraploid *Th. elongatum* confirmed that 21, 106, 50, and 73 markers can detect E genome chromosomes in diploid *Th. elongatum*, *Th. ponticum*, *Tr. caespitosum* (StE), and *Psa. athericum* (StEP), respectively (see Additional file 2: Table S2). Our results provide further evidence that tetraploid *Th. elongatum* is an allotetraploid harboring two different E genomes, and *Thinopyrum* E genomes were differentiated during polyploidization events. Two specific markers, TTE1E-212 and TTE1E-222, were unique to diploid *Th. elongatum*, tetraploid *Th. elongatum*, and *Th. ponticum*, suggesting they can detect the E chromosomal DNA in all materials carrying the E genome. Interestingly, 58, 32, 5, 8, 28, 8, and 13 markers based on specific tetraploid *Th. elongatum* sequences successfully amplified products from *Th. bessarabicum* (E^b^), *Pse. libanotica* (St), *D. villosum* (V), *H. bogdanii* (H), *Ag. cristatum* (P), *S. cereale* (R), and *P. huashanica* (Ns), respectively. Our specificity analysis indicated that the marker amplification frequencies are considerably higher in E, E^b^, and St genomic species than in other wheat-related species, which is consistent with the findings of previous studies, in which the E genomes of *Th. elongatum* and *Th. ponticum* were more closely related to the E^b^ genome of *Th. bessarabicum* and the St genome of *Pseudoroegneria* species than to the other genomes [33, 46]. Additionally, 48 and 74 markers uncovered polymorphisms between *Thinopyrum* E-genome-containing species and *Th. bessarabicum* and *Pse. libanotica*, respectively, indicating that they will likely be applicable for investigating the genetic differences between E, E^b^, and St genomes and for revealing the genetic diversity among species (populations) carrying E, E^b^, and/or St genomes. The resulting information will further characterize these complex species and may be relevant for the genetic improvement of these important forage crops.

## Conclusions

We characterized a wheat-tetraploid *Th. elongatum* 1E (1D) disomic substitution line based on GISH, FISH, and SSR analyses. This line is highly resistant to the stripe rust pathogen strains prevalent in China. Moreover, it represents an appropriate bridge breeding material for the introgression of alien genes to improve wheat disease resistance. Additionally, we analyzed GBS data to generate and validate a new 1E chromosome-specific, easy-to-use marker set that may be applicable for identifying and characterizing the tetraploid *Th. elongatum* chromosomes along with the chromosomes of all other wheat-related species and for investigating the genetic differences and phylogenetic relationships among E, E^b^, St, and other closely-related genomes. The use of this marker set will further increase our understanding of these complex species.

## Materials and methods

### Plant materials

**Fig. 11:**
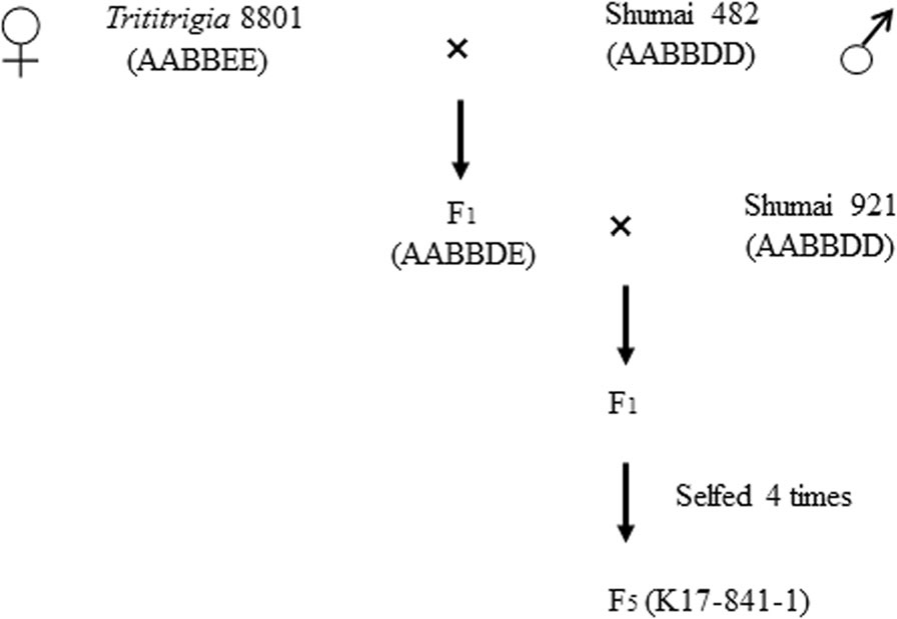
Pedigree details of K17–841-1

The plant materials used in the current study are listed in Table 5. The hexaploid *Trititrigia* line 8801 (2*n* = 6*x* = 42, AABBEE), which is tolerant to cold, drought, and salt stresses and resistant to Fusarium head blight, rust, and powdery mildew, was originally produced and identified at the Eastern Cereal and Oilseed Research Center, Ottawa, Canada [17]. The native wheat cultivars SM482, SM921, and SM969 exhibit superior agronomic traits, but are susceptible to the stripe rust pathogens prevalent in southwestern China. To produce wheat–tetraploid *Th. elongatum* derivative line, we first crossed 8801 with SM482, after which the resulting F_1_ plants were further crossed with SM921 to obtain the BC_1_F_1_ population. Seeds from the BC_1_F_1_ plants were bulked and advanced to the BC_1_F_5_ generation by single seed descent, ultimately resulting in the isolation of line K17–841-1 (Fig. 11). The F_2_ population comprising 80 individuals was derived from a cross between K17–841-1 and SM969. We developed six wheat–tetraploid *Th. elongatum* disomic substitution lines, TDS2E (2A), TDS3E (3D), TDS4E (4D), TDS5E (5D), TDS6E (6D), and TDS7E (7D), via the hybridization between native wheat cultivars and *Trititrigia* line 8801 (data not published). Wheat cultivar Chinese Spring (CS) was used as a positive control for the molecular marker analysis. Wheat line SY95–71 was used as a susceptible control for the evaluations of stripe rust responses. Regarding the GISH analysis, wheat cultivar J-11 was used as a source of blocking DNA, whereas tetraploid *Th. elongatum* accession PI531750 (2*n* = 4*x* = 28, EEEE) was used as a source of probe DNA. The molecular markers were validated with the following wheat-related species: *Thinopyrum elongatum* (2*n* = 2*x* = 14, EE), *Thinopyrum ponticum* (2*n* = 10*x* = 70, EEEEEEEEEE/StStStStEEEEEE), *Thinopyrum bessarabicum* (2*n* = 2*x* = 14, E^b^E^b^), *Pseudoroegneria libanotica* (2*n* = 2*x* = 14, StSt), *Dasypyrum villosum* (2*n* = 2*x* = 14, VV), *Hordeum bogdanii* (2*n* = 2*x* = 14, HH), *Agropyron cristatum* (2*n* = 2*x* = 14, PP), *Secale cereale* (2*n* = 2*x* = 14, RR), *Psathyrostachys huashanica* (2*n* = 2*x* = 14, NsNs), *Trichopyrum caespitosum* (2*n* = 4*x* = 28, StStEE), and *Psammopyrum athericum* (2*n* = 6*x* = 42, StStEEPP). Voucher specimens have been deposited in the herbarium of the Triticeae Research Institute, Sichuan Agricultural University, China (SAUTI).

**Table 5:** Plant materials used in this study

Accession	Species/Materials	Chromosome numbers	Genome
PI531718	*Thinopyrum elongatum*	14	E
PI531750	*Thinopyrum elongatum*	28	EE
PI531737	*Thinopyrum ponticum*	70	EEEEE/StStEEE
W6–10232	*Thinopyrum bessarabicum*	14	E^b^
PI228391	*Pseudoroegneria libanotica*	14	St
PI251477	*Dasypyrum villosum*	14	V
Y1819	*Hordeum bogdanii*	14	H
PI610892	*Agropyron cristatum*	14	P
QL	*Secale cereale*	14	R
ZY3156	*Psathyrostachys huashanica*	14	Ns
PI634264	*Trichopyrum caespitosum*	28	StE
PI531744	*Psammopyrum athericum*	42	StEP
8801	*Triticum durum*-tetraploid *Thinopyrum elongatum* amphidiploid	42	ABE
SM482	Shumai482	42	ABD
SM921	Shumai921	42	ABD
SM969	Shumai969	42	ABD
CS	Chinese Spring	42	ABD
J-11	wheat cultivar J-11	42	ABD
SY95–71	wheat line SY95–71	42	ABD
K17–841-1	Wheat-tetraploid *Thinopyrum elongatum* derivative line	42	ABDE
TDS2E(2A),TDS3E(3D), TDS4E(4D),TDS5E(5D), TDS6E(6D),TDS7E(7D)	Wheat-tetraploid *Thinopyrum elongatum* disomic substitution lines	42	ABDE
	F_2_ population of K17–841-1/Shumai969	42

### GISH and FISH analyses

Root tips from germinating seeds were treated with nitrous oxide for 2 h and 90% acetic acid for 10 min, after which they were digested with pectinase and cellulase (Yakult Pharmaceutical, Tokyo, Japan) [47]. Slides were then prepared for the GISH analysis as described by Han et al. [48]. The cetyltrimethyl ammonium bromide method was used to extract genomic DNA from the freshly collected leaves of tetraploid *Th. elongatum* PI531750 and wheat cultivar J-11 [49]. The PI531750 DNA was labeled with fluorescein-12-dUTP according to the nick translation method (Thermo Fisher Scientific, Eugene, OR, USA) and was used as the hybridization probe. The GISH analysis was completed according to the method described by Han et al. [50], with a 1:150 probe DNA:blocking DNA ratio. Specifically, 10 μL hybridization solution containing 2 × saline sodium citrate (SSC), 10% dextran sulfate, and 10 ng/μL labeled probe DNA together with blocking DNA was added to each slide. Samples were denatured by heating at 85 °C for 5 min, incubated at 37 °C for 8 h, and washed with 2× SSC. Finally, the chromosomes were counterstained with 4,6-diamino-2- phenylindole solution (Vector Laboratories, Burlingame, CA, USA). The GISH slides were examined with the BX-63 microscope (Olympus, Tokyo, Japan) and images were captured with a DP-70 CCD camera.

The GISH slides were washed sequentially with 70% (v/v) ethanol for 5 min, 2× SSC at 60 °C for 30 min, ddH_2_O for 10 min, and 100% (v/v) ethanol for 5 min. A FISH analysis was subsequently completed to identify the chromosomal constitution of line K17–841-1, with pSc119.2 and pTa535 as probes [47, 51]. The FISH was performed as described by Han et al. [48], with minor modifications. The probe mixture (0.35 μL each probe in 2× SSC and 1× TE buffer, pH 7.0; total volume = 10 μL) was added to a slide, covered with a coverslip, incubated in a moist box at 37 °C for 2 h, and then washed with 2× SSC at room temperature. The FISH signals were observed with the BX63 microscope (Olympus). Images were captured with the DP-70 CCD camera and analyzed with Adobe Photoshop software.

### SSR marker analysis

Primer pairs for four SSR markers (*wmc147*, *wmc222*, *gwm337*, and *Xcfd63*) on wheat chromosomes 1DS and 1DL were used to characterize the 1D chromatin in the wheat–tetraploid *Th. elongatum* line K17–841-1. Details regarding all primers are listed in Table 6. Additionally, CS, SM482, and SM921 were used as positive controls, whereas 8801 was used as a negative control. The PCR amplification was completed as described by Somers et al. [52], with minor modifications.

**Table 6:** Sequences of wheat 1DS and 1DL SSR markers

Marker	Primer sequence (5′-3′)	Annealing temperature (°C)	Arm location	Amplification size (bp)
*wmc147*	F: AGAACGAAAGAAGCGCGCTGAG R: ATGTGTTTCTTATCCTGCGGGC	58	1DS	154
*wmc222*	F: AAAGGTGCGTTCATAGAAAATTAGA R:AGAGGTGTTTGAGACTAATTTGGTA	54	1DS	184
*gwm337*	F: CCTCTTCCTCCCTCACTTAGC R: TGCTAACTGGCCTTTGCC	59	1DL	191
*Xcfd63*	F: TCCTGAGGATGTTGAGGACC R: GAGAGAGGCGAAACATGGAC	58	1DL	282

*F* forward primer, *R* reverse primer

### Agronomic trait evaluation

The morphological traits of K17–841-1 and its parents were evaluated in a field trial in Wenjiang, Sichuan province, China, with three replicates in the 2017–2018 and 2018–2019 growing seasons. For each replicate, 15 grains of each line were evenly planted in 1.5-m rows separated by 0.3 m. The plant height, tiller number, spike length, spikelet per spike, grains per spike, thousand-grain weight, and seed setting rate were evaluated for 10 samples per replicate. Significant differences in traits were determined with the SAS 8.2 program (SAS Institute Inc., Cary, NC, USA).

### Stripe rust resistance screening

The smear method [53] was used to evaluate the responses of adult K17–841-1, 8801, SM482, SM921, and SY95–71 plants to a mixture of *Pst* races (CYR-32, CYR-33, CYR-34, and V26/Gui22–14) in a field trial in Chengdu, Sichuan, China during the 2017–2018 growing season. To assess the utility of the developed tetraploid *Th. elongatum* 1E chromosome-specific markers, the K17–841-1/SM969 F_2_ individuals as well as 8801, SM482, SM921, SM969, and SY95–71 seedling were evaluated for their reactions to *Pst* race CYR-34 in a growth chamber. The plants were inoculated at the two-leaf stage and the stripe rust reaction of the first leaf of each plant was evaluated at 14 days after inoculation. Wheat line SY95–71 was used as the susceptible control. The stripe rust responses were evaluated with three replicates as described by Li et al. [17]. The stripe rust IT was based on the following scale: 0, 0;, 1, 2, 3, and 4, in which 0 = immunity, 0; = necrotic flecks, and 1–4 = increasing sporulation and decreasing necrosis or chlorosis. Plants with an IT of 2 or lower were considered resistant, whereas plants with an IT of 3 or 4 were considered susceptible.

### Genotyping-by-sequencing, sequence alignment, and tetraploid *Th. elongatum* 1E chromosome– specific fragment acquisition

Total genomic DNA was extracted from fresh young leaves with the cetyltrimethyl ammonium bromide method, after which the DNA concentration was adjusted to 150 ng/μL. Genomic DNA samples of diploid *Th. elongatum* PI531718, PI531750, 8801, K17–841-1, and six wheat–tetraploid *Th. elongatum* (2E-7E) chromosomal substitution lines were subjected to GBS, which was completed by Novogene Bioinformatics Technology Co., Ltd. (Beijing, China). High-Quality DNA libraries were constructed and sequenced with the Illumina HiSeq™ system. The raw reads in FASTQ format were filtered by removing low-quality reads and reads with adapter and/or poly-N sequences to obtain clean reads (140 bp). Tetraploid *Th. elongatum* 1E chromosome-specific sequences were obtained as follows. First, the high-quality K17–841-1 sequences were compared with the CS reference genome sequences (*https://urgi.versailles.inra.fr/download/iwgsc/IWGSC_RefSeq_Assemblies/v1.0/*), after which the sequences with identities greater than 23% were eliminated [54]. Second, the sequences were compared with those of 8801 and PI531750 acquired by GBS in this study, and the sequences with identities greater than 23% were selected. Finally, the retained K17–841-1 sequences were compared with the sequences of PI531718 and the six chromosomal substitution lines. Sequences with identities exceeding 90% were removed. The remaining sequences were considered to be specific to chromosome 1E of tetraploid *Th. elongatum*.

### Development and validation of the tetraploid *Th. elongatum*-specific molecular markers

On the basis of these specific sequences, PCR primers were designed with the Primer3 Plus online tool (http://www.bioinformatics.nl/cgi-bin/primer3plus/primer3plus.cgi) and then synthesized by TSINGKE (Chengdu, China). Details regarding the PCR primers are presented in Additional file 1: Table S1. The amplified products were examined by 3% agarose electrophoresis. The markers that amplified specific sequences in PI531750, 8801, and K17–841-1, but not in SM482, SM921, CS, PI531718, and the 2E-7E substitution lines, were identified as tetraploid *Th. elongatum* 1E chromosome-specific molecular markers. The stability, repeatability, and specificity of these markers were validated in the CS, PI531750, 8801, and K17–841-1 lines as well as in 11 wheat-related species.

The PCR amplifications were completed in a reaction volume of 25 μL, which included 1.0 μL template DNA (100 ng/μL), 12.5 μL 2× Taq Master Mix for PAGE (Dye plus), 1.0 μL each primer (10 μM), and 9.5 μL ddH_2_O. The PCR program was as follows: 94 °C for 5 min; 35 cycles of 94 °C for 30 s, appropriate annealing temperature of 50–60 °C for 30 s, and 72 °C for 1 min; 72 °C for 10 min.

## Supplementary information

**Additional file 1:** **Table S1.** PCR amplification results of tetraploid *Th. elongatum* specific molecular markers.

**Additional file 2:** **Table S2.** Specific amplification of 1E chromosome markers in wheat-related species.

**Additional file 3:** **Table S3.** Stripe rust response at seedling stage and specific amplification of 1E chromosome markers in F_2_ population.

## Data Availability

The datasets generated and analyzed during the present study are available from the corresponding author on reasonable request.

## Abbreviations

CS: Chinese Spring
FISH: Fluorescence in situ hybridization
GBS: Genotyping-by-sequencing
GISH: Genome in situ hybridization
MAS: Marker-assisted selection
PCR: Polymerase chain reaction
*Pst*: *Puccinia striiformis* f. sp*. tritici*
SM482: Shumai482
SM921: Shumai921
SM969: Shumai969
SSR: Simple sequence repeats
