# Supplementary material for: Characterization of a wheat–tetraploid Thinopyrum elongatum 1E(1D) substitution line K17–841-1 by cytological and phenotypic analysis and developed molecular markers
Source: BMC Genomics. 2019 Dec 10;20:963. doi: 10.1186/s12864-019-6359-9 (PMC6905003; doi:10.1186/s12864-019-6359-9)
Supplement: Supplementary file 1 — Additional file 1: Table S1. PCR amplification results of tetraploid Th. elongatum specific molecular markers. [file 12864_2019_6359_MOESM1_ESM.docx]

**Table S1** PCR amplification results of tetraploid *Th. elongatum* molecular markers

| Specific Markers | Specific Primers | DNA Sequences of the Special Primers (5'–3') | | Amplified Chromosomes | Annealing Temperatures | Fragments Size | Types |
| --- | --- | --- | --- | --- | --- | --- | --- |
|  |  | Forward | Reverse |  |  |  |  |
| TTE1E-1 | Primer1E-1 | AAACCCGCAGATGTTGGTAG | TTTTGCAGTAGCGTCCACTC | 1E | 55 | 197 | Ⅱ |
| TTE1E-2 | Primer1E-2 | GCAGCGAATGTAGGTAGTGAGA | GCCACAGATTCATCCCTGA | 1E | 56 | 97 | Ⅱ |
| TTE1E-3 | Primer1E-3 | GCATAGCCAATCGGACAGTA | CTGCCGAAACCAAGGAGA | 1E, 5E, 6E | 55 | 191 | Ⅲ |
| TTE1E-4 | Primer1E-4 | GTTGTGTCCGCCCTCAAT | CGGTTACCAATGGTCGTTAC | 1E | 55 | 168 | Ⅰ |
| TTE1E-5 | Primer1E-5 | GTGGAGGAGCGACGATAGAT | CTAGCTAGGCTGTGCGTGTG | 1E | 58 | 206 | Ⅰ |
| TTE1E-6 | Primer1E-6 | AAGTGGCCAGGTCGACAG | TTTAGTACTGGGTCGGACTGC |  | 60 | 105 | Ⅳ |
| TTE1E-7 | Primer1E-7 | GGTCGTCAACATTCCAGTGA | CCCAACAATCCCATCTCG | 1E | 55 | 219 | Ⅰ |
| TTE1E-8 | Primer1E-8 | CTCACCTTGCTGGCCAAT | GAAATCAAGGGTGCTGCTTC | 1E | 55 | 46 | Ⅰ |
| TTE1E-9 | Primer1E-9 | GGTGACCCTCTATCACCCTTT | ATCACCAGGCAAGGAAGAGA | 1E | 57 | 146 | Ⅰ |
| TTE1E-10 | Primer1E-10 | TGAAGGAAGCCCCTCAAA | GGCTGAAGTGATTGAGCTG | 1E | 54 | 106 | Ⅱ |
| TTE1E-11 | Primer1E-11 | GGGTGGGCTTTTCAGTTTAC | CAATCCCCAATCACCCATAC |  | 53 | 194 | Ⅴ |
| TTE1E-12 | Primer1E-12 | GCCAGGAAATGGTTAGAAGG | CTTCCTTGGGGACTAACATACG | 1E | 57 | 213 | Ⅰ |
| TTE1E-13 | Primer1E-13 | CTATCGGGGTAGGTGTTTGC | ACTCAGGCCCAATCCCTAGT | 1E | 57 | 90 | Ⅰ |
| TTE1E-14 | Primer1E-14 | TCGTAAGTGGGTCTACGTGCT | AGCACCCCAATCGTATCAAG | 1E | 57 | 86 | Ⅰ |
| TTE1E-15 | Primer1E-15 | GCCGAGTCCCTGATAGAATG | CGGATTTACGTCGCACAA | 1E | 55 | 219 | Ⅰ |
| TTE1E-16 | Primer1E-16 | GGCATTCGTTTGTGTGAGTC | CTAAGAACAAGGCGGTTGTG | 1E | 55 | 172 | Ⅰ |
| TTE1E-17 | Primer1E-17 | GCGAAACCACCGGTTATCTA | GAGCACTTCGCTTCGTTACTC | 1E | 57 | 82 | Ⅰ |
| TTE1E-18 | Primer1E-18 | GGCCCATTTTACACTGCTCT | CACTAGGAGCTCCCATCCTG |  | 57 | 67 | Ⅳ |
| TTE1E-19 | Primer1E-19 | ACAGACCCCACAACATTCCT | GGCAGTTGAACCTTTCCA | 1E | 54 | 95 | Ⅰ |
| TTE1E-20 | Primer1E-20 | TAGGTGGCTGCTGCTTCTC | GCAGCCGTTGTTTTTGCT | 1E | 55 | 114 | Ⅰ |
| TTE1E-21 | Primer1E-21 | TGTGCCACTACCTGAACTCG | ATGCCGAGTCTGTTGTCTTG | 1E | 56 | 198 | Ⅰ |
| TTE1E-22 | Primer1E-22 | CCCTTAGGAAAGGAGCTTCA | TTCGTTCTGCTCGTCTTCAC | 1E | 55 | 182 | Ⅰ |
| TTE1E-23 | Primer1E-23 | TTGCTAAGTGTGGGAGAGGA | CCCCTGCTCTTACCAAATG | 1E | 55 | 100 | Ⅰ |
| TTE1E-24 | Primer1E-24 | AATGACCCTTTGGGTAGTGG | GGCGCGAAAAGGATTGTA |  | 54 | 192 | Ⅳ |
| TTE1E-25 | Primer1E-25 | ATCGCGTCCTAGTCAGCATT | CGGGACCGAGAGGAGTATAA | 1E | 56 | 72 | Ⅱ |
| TTE1E-26 | Primer1E-26 | CAACCGACCAGTCTTTCCA | CCCACAGAATGTAGCCCACT | 1E | 56 | 110 | Ⅰ |
| TTE1E-27 | Primer1E-27 | TTTTGCGGATCGGTTAGG | TTGCCGCTTGTAGTGTGTTC | 1E | 54 | 114 | Ⅱ |
| TTE1E-28 | Primer1E-28 | ACTATGGGTTGGCTGGATGT | CCTCCACCGATATTCCAATC | 1E | 55 | 109 | Ⅰ |
| TTE1E-29 | Primer1E-29 | TGGTGACTTGATCCTCCCTA | GCTTGAGAGCATTAGCCTTG | 1E | 55 | 200 | Ⅰ |
| TTE1E-30 | Primer1E-30 | TCGTAAGTGGGTCTACGTGCT | AGCACCCCAATCGTATCAAG | 1E | 57 | 86 | Ⅰ |
| TTE1E-31 | Primer1E-31 | GCCCTGTTTGTACGGGATTA | GATCGCCACACCAACTGA | 1E | 55 | 171 | Ⅰ |
| TTE1E-32 | Primer1E-32 | ACCACCCACTCAAACCACTT | ATGGACCCCCAATCCTTT |  | 54 | 119 | Ⅳ |
| TTE1E-33 | Primer1E-33 | CAACCTGAAACCACAACTCC | CGCGCGTTAGTGATGATAC | 1E | 55 | 111 | Ⅰ |
| TTE1E-34 | Primer1E-34 | CCACCGCAATTCAAACAC | TTCAGGGCATCTCCCTAGTT |  | 54 | 108 | Ⅳ |
| TTE1E-35 | Primer1E-35 | TGCTCCAAACCAAACCAG | TGCGATCAACACTGGGTTAC | 1E | 54 | 99 | Ⅰ |
| TTE1E-36 | Primer1E-36 | CGACCAACGGGTCAAGTAA | CGACTTCCCTTGTCTGAACTG |  | 53 | 106 | Ⅴ |
| TTE1E-37 | Primer1E-37 | GGATCGTCGACCCTCTTTT | CTGAAAGCAGCACATCACTG |  | 55 | 97 | Ⅳ |
| TTE1E-38 | Primer1E-38 | CCCTGACGACAAGATATGGAG | AGGCCAAGGAGTGAATGGTA |  | 57 | 114 | Ⅳ |
| TTE1E-39 | Primer1E-39 | TGCTGACAACCTACCACAGG | TGGTTTGGACTTGAGGGTTC | 1E | 56 | 186 | Ⅰ |
| TTE1E-40 | Primer1E-40 | CTGCCGAAACCAAGGAGA | GCATAGCCAATCGGACAGTA | 1E, 5E, 6E | 55 | 190 | Ⅲ |
| TTE1E-41 | Primer1E-41 | ATGTCTACCCTCGGGTCAAG | CGACTTCCCTTGTCTGAACTG | 1E | 58 | 110 | Ⅱ |
| TTE1E-42 | Primer1E-42 | CTGAAAGCAGCACATCACTG | GGATCGTCGACCCTAGTTTG |  | 56 | 97 | Ⅳ |
| TTE1E-43 | Primer1E-43 | TGTACCCGTAGCTTCTTTGG | GGAAGGCTGGAGGAGGAC | 1E, 7E | 57 | 91 | Ⅲ |
| TTE1E-44 | Primer1E-44 | CCACGAGGTAGCAACAGAGA | GGGCCAGTAGGTCAACACAT | 1E | 57 | 110 | Ⅰ |
| TTE1E-45 | Primer1E-45 | GCTAGGGCCCCATTTCATA | CCTCTAGAGATCCATATCCTCTGC | 1E | 57 | 83 | Ⅰ |
| TTE1E-46 | Primer1E-46 | GGCAGTTGAACCTTTCCA | ACAGACCCCACAACATTCCT | 1E | 54 | 95 | Ⅰ |
| TTE1E-47 | Primer1E-47 | AACACCCCCTAAAGGCAACT | AGAGGTGGCTGCGGATACT | 1E | 56 | 112 | Ⅰ |
| TTE1E-48 | Primer1E-48 | ATGGCCTGCTTGGTAAGCTA | GCGTGGGGAGAAGTTCAG |  | 56 | 116 | Ⅳ |
| TTE1E-49 | Primer1E-49 | TTTTGCAGTAGCGTCCACTC | AAACCCGCAGATGTTGGTAG | 1E | 55 | 197 | Ⅱ |
| TTE1E-50 | Primer1E-50 | TCCCACCTCACTTCCTTACA | GGACTAATTTGGCCCCTCA | 1E | 55 | 220 | Ⅱ |
| TTE1E-51 | Primer1E-51 | CCTCTAGAGATCCATATCCTCTGC | GCTAGGGCCCCATTTCATA | 1E | 57 | 83 | Ⅰ |
| TTE1E-52 | Primer1E-52 | AAGTGGCCAGGTCGACAG | TTTAGTACTGGGTCGGACTGC |  | 57 | 105 | Ⅳ |
| TTE1E-53 | Primer1E-53 | GCTTGAGAGCATTAGCCTTG | TGGTGACTTGATCCTCCCTA | 1E | 55 | 200 | Ⅰ |
| TTE1E-54 | Primer1E-54 | CTGTGAGCAATAACGCAAGG | CCTTACTCCTCTTGCCATCC | 1E | 56 | 186 | Ⅱ |
| TTE1E-55 | Primer1E-55 | CTCTCCCGGAGTACAAGTGC | AAGGAGATGTAGCACGTAGGAG | 1E | 59 | 216 | Ⅰ |
| TTE1E-56 | Primer1E-56 | ATTTGGGTCCATGGGTGA | TGGTTTGGACTTGAGGGTTC | 1E | 54 | 113 | Ⅰ |
| TTE1E-57 | Primer1E-57 | GGCTGAAGTGATTGAGCTG | TGAAGGAAGCCCCTCAAA | 1E | 54 | 106 | Ⅱ |
| TTE1E-58 | Primer1E-58 | GCCGCTTACCTGTCACTGTA | TAAGTGGGGAGATGCTAGGG | 1E, 2E, 6E, 7E | 57 | 116 | Ⅲ |
| TTE1E-59 | Primer1E-59 | CCTTACTCCTCTTGCCATCC | CTGTGAGCAATAACGCAAGG | 1E | 56 | 186 | Ⅱ |
| TTE1E-60 | Primer1E-60 | ATGCCGAGTCTGTTGTCTTG | TGTGCCACTACCTGAACTCG | 1E | 56 | 198 | Ⅰ |
| TTE1E-61 | Primer1E-61 | ACAACCTGACCCCACAACTC | CGCGCGTTAGTGATGATAC | 1E | 56 | 112 | Ⅰ |
| TTE1E-62 | Primer1E-62 | TGGTTTGGACTTGAGGGTTC | GGCGACCTACCACAGGAA | 1E | 56 | 182 | Ⅰ |
| TTE1E-63 | Primer1E-63 | GCAGTAGCGCATAGCCAAT | CTGCCGAAACCAAGGAGA | 1E, 5E, 6E | 55 | 198 | Ⅲ |
| TTE1E-64 | Primer1E-64 | GGGTCAAGTCCCCTAGAGC | TAGGAAGGTAGCTCCCAACG | 1E | 58 | 116 | Ⅰ |
| TTE1E-65 | Primer1E-65 | CAACCGACCAGTCTTTCCA | CCACAGAATGTAGCCCACTC | 1E | 56 | 109 | Ⅰ |
| TTE1E-66 | Primer1E-66 | ACGGGTCAATTCACCAAGAG | TAGGAAGGTAGCTCCCAACG |  | 54 | 118 | Ⅴ |
| TTE1E-67 | Primer1E-67 | GCCAGGTGAGTTGCTGTTC | AAACTGTTAGGAGGCCCACA |  | 58 | 74 | Ⅳ |
| TTE1E-68 | Primer1E-68 | CGACTTCCCTTGTCTGAACTG | ACCATCGGTTCAAGTCACCT |  | 57 | 105 | Ⅳ |
| TTE1E-69 | Primer1E-69 | TGCGACTGAGACTAGCTTGG | AAGCGTGAGCCAGCAAAT | 1E | 55 | 204 | Ⅰ |
| TTE1E-70 | Primer1E-70 | GGCCAGTAGGTCACCACATT | CCACGAGGTAGCAACAGAGA | 1E | 57 | 109 | Ⅰ |
| TTE1E-71 | Primer1E-71 | GGGCCAGTAGGTCAACACAT | CCACGAGGTAGCAACAGAGA | 1E | 57 | 110 | Ⅰ |
| TTE1E-72 | Primer1E-72 | TGGTGGATCGAGTCGTTTAG | AAGTGGCCAGGTCGACAG |  | 56 | 120 | Ⅳ |
| TTE1E-73 | Primer1E-73 | AAGTGGCCAGGTCGACAG | TTTAGTACTGGGTCGGACTGC |  | 59 | 105 | Ⅳ |
| TTE1E-74 | Primer1E-74 | CACATCTCTCACAGCATCACAC | CTGGGCGACACATTATTGG | 1E, 2E, 5E | 56 | 144 | Ⅲ |
| TTE1E-75 | Primer1E-75 | ACCTACCACCGGAAAGGAAT | TGGTTTGGACTTGAGGGTTC | 1E | 55 | 178 | Ⅰ |
| TTE1E-76 | Primer1E-76 | CCCCGGAGATAGAGGTAGC | TTGAAAGCGTCCACGAGTC |  | 55 | 101 | Ⅴ |
| TTE1E-77 | Primer1E-77 | CTAAGAACAAGGCGGTTGTG | GGCATTCGTTTGTGTGAGTC | 1E | 55 | 172 | Ⅰ |
| TTE1E-78 | Primer1E-78 | GGTGACCCTCTATCACCCTTT | ATCACCAGGCAAGGAAGAGA | 1E | 57 | 146 | Ⅰ |
| TTE1E-79 | Primer1E-79 | GTGTGTAAGCCATCGGAGGT | TGACCTGTTCACTGCGAAAC | 1E, 2E, 6E | 56 | 206 | Ⅲ |
| TTE1E-80 | Primer1E-80 | GGGCCAGTAGGTCAACACAT | CCACGAGGTAGCAACAGAGA | 1E | 57 | 110 | Ⅰ |
| TTE1E-81 | Primer1E-81 | CTCACCATCTCTCTCCAATCTG | GAAGGTGGCAGCTTGTGTAT | 1E | 57 | 120 | Ⅱ |
| TTE1E-82 | Primer1E-82 | TCACCTTTCTGGCCAAGC | GAAATCAAGGGTGCTGCTTC | 1E | 55 | 45 | Ⅰ |
| TTE1E-83 | Primer1E-83 | GGCATTCGTTTGTGTGAGTC | CACCTTCTTGGTCAGAGCAT | 1E | 55 | 108 | Ⅰ |
| TTE1E-84 | Primer1E-84 | CACCAGGCAAGGAAGAGAAT | GGTGACCCTCTATCACCCTTT | 1E | 57 | 144 | Ⅰ |
| TTE1E-85 | Primer1E-85 | TCTATGCAGTGATCGAGACG | CCAGGAGCCAAAAATCCTC | 1E | 55 | 194 | Ⅰ |
| TTE1E-86 | Primer1E-86 | TTTAGTACTGGGTCGGACTGC | AAGTGGCCAGGTCGACAG |  | 59 | 105 | Ⅳ |
| TTE1E-87 | Primer1E-87 | TTTAGTACTGGGTCGGACTGC | AAGTGGCCAGGTCGACAG |  | 59 | 105 | Ⅳ |
| TTE1E-88 | Primer1E-88 | CGATGTTGCACGTACCCTAA | GGACTCGCTGAAGTCGAAAT | 1E | 55 | 95 | Ⅰ |
| TTE1E-89 | Primer1E-89 | GGAGCCCTCAAATCCTATTG | CCTTCGCTCCTCTTTGATTC | 1E | 55 | 118 | Ⅰ |
| TTE1E-90 | Primer1E-90 | CAGCCGGGTATTCAATGG | CGGCTGTAAAGTCCCACTTC | 1E | 56 | 97 | Ⅰ |
| TTE1E-91 | Primer1E-91 | CTTCTCAGTCTGTTGCAGGTG | GGCCCTTTTTGTAGTGTGGA | 1E | 55 | 85 | Ⅰ |
| TTE1E-92 | Primer1E-92 | TCAAGCGCTTAGTAGACCACTC | AGCACGGGATAACTTGAACC | 1E | 57 | 187 | Ⅰ |
| TTE1E-93 | Primer1E-93 | TCAAGCGCTTAGTAGACCACTC | CTTGAACCCAGTTGCGAAG | 1E | 56 | 175 | Ⅰ |
| TTE1E-94 | Primer1E-94 | GAAGGAAGCCCCTCAAATG | GGCTGAAGTGATTGAGCTG |  | 55 | 105 | Ⅴ |
| TTE1E-95 | Primer1E-95 | TGCTGTCAAAGCGAAAGC | GAAATCAAGGGTGCTGCTTC | 1E | 54 | 39 | Ⅰ |
| TTE1E-96 | Primer1E-96 | TGTGCCACTACCTGAACTCG | ATGCCGAGTCTGTTGTCTTG | 1E | 56 | 198 | Ⅰ |
| TTE1E-97 | Primer1E-97 | TGGCCACTAGGTATCAACAGAG | GGGCCAGTAGGTCAACACAT | 1E | 58 | 113 | Ⅰ |
| TTE1E-98 | Primer1E-98 | GCGTGGGGAGAAGTTCAG | ATGGCCTGCTTGGTAAGCTA |  | 56 | 117 | Ⅳ |
| TTE1E-99 | Primer1E-99 | TGAGCCTATAGCTGCTGGAA | CGTCCCACCTCCCTTTTT | 1E | 55 | 167 | Ⅰ |
| TTE1E-100 | Primer1E-100 | TTCGTTCTGCTCGTCTTCAC | CCCTTAGGAAAGGAGCTTCA | 1E | 55 | 182 | Ⅰ |
| TTE1E-101 | Primer1E-101 | CACGGGCAATCAGCTAGTAA | TTCGTTCTGCTCGTCTTCAC | 1E | 55 | 260 | Ⅰ |
| TTE1E-102 | Primer1E-102 | GCATAGCCAATCGGACAGTA | GGCCTGCTAGTGCACTTTTT | 1E, 5E, 6E | 55 | 117 | Ⅲ |
| TTE1E-103 | Primer1E-103 | GGCAGTTGAACCTTTCCA | ACAGACCCCACAACATTCCT | 1E | 54 | 95 | Ⅰ |
| TTE1E-104 | Primer1E-104 | GGCAGTTGAACCTTTCCA | ACAGACCCCACAACATTCCT | 1E | 54 | 95 | Ⅰ |
| TTE1E-105 | Primer1E-105 | AAAGCCGGATTAGCTCCTTC | GAAGCTTGCATCAGGGTCTT | 1E, 6E | 55 | 219 | Ⅲ |
| TTE1E-106 | Primer1E-106 | TGCTGACAACCTACCACAGG | TGGTTTGGACTTGAGGGTTC |  | 56 | 186 | Ⅳ |
| TTE1E-107 | Primer1E-107 | AAGGAGATGTAGCACGTAGGAG | CTCTCCCGGAGTACAAGTGC | 1E | 59 | 216 | Ⅰ |
| TTE1E-108 | Primer1E-108 | ACAGACCCCACAACATTCCT | GGCAGTTGAACCTTTCCA | 1E | 54 | 95 | Ⅰ |
| TTE1E-109 | Primer1E-109 | CCACTCGTAAGTGGGTCTACG | AGCACCCCAATCGTATCAAG |  | 57 | 90 | Ⅳ |
| TTE1E-110 | Primer1E-110 | CCACGAGGTAGCAACAGAGA | GGGCCAGTAGGTCAACACAT | 1E | 57 | 110 | Ⅰ |
| TTE1E-111 | Primer1E-111 | ACAACCTGACCCCACAACTC | CGCGCGTTAGTGATGATAC | 1E | 56 | 112 | Ⅰ |
| TTE1E-112 | Primer1E-112 | TACGTACGTGATCGGTCGAG | CATCACCCGACACGAGATTA |  | 56 | 101 | Ⅳ |
| TTE1E-113 | Primer1E-113 | CGCACCAAAGACATCTCG | AGTCATTTCACGCGTCGTC | 1E | 55 | 105 | Ⅰ |
| TTE1E-114 | Primer1E-114 | TCTGGATCGTCGACCCTATT | CTGAAAGCAGCACATCACTG |  | 55 | 100 | Ⅳ |
| TTE1E-115 | Primer1E-115 | TGTCACTTCGAGGCTGATTG | TCGTCAAACGTTGGCATC | 1E | 54 | 43 | Ⅱ |
| TTE1E-116 | Primer1E-116 | GGGGAGTCACTGCATGGT | AAAGTGGTCTGGTCCGATTG |  | 56 | 175 | Ⅴ |
| TTE1E-117 | Primer1E-117 | CGCTTGAACCTGTCAATACC | CGCAGGTGTAGGGATCAAA | 1E | 55 | 75 | Ⅰ |
| TTE1E-118 | Primer1E-118 | TTCAGGGCATCTCCCTAGTT | CCACCGCAATTCAAACAC |  | 54 | 108 | Ⅳ |
| TTE1E-119 | Primer1E-119 | TACATTAGACGCGTGGAGGT | GTTGCAAGGCGAGAAAGG |  | 55 | 70 | Ⅳ |
| TTE1E-120 | Primer1E-120 | TGCAGAGGATGCGTGTGTA | TCGTCAAACGTTGGCATC | 1E | 54 | 118 | Ⅰ |
| TTE1E-121 | Primer1E-121 | CACAGTTCCGTAGGTGCAGA | GCTCACCATTGCAGTTGAAG | 1E | 56 | 109 | Ⅰ |
| TTE1E-122 | Primer1E-122 | GACCGAGTGCTACCCAGATT | GGATTTCCACCAGTGCCTAT | 1E | 56 | 154 | Ⅰ |
| TTE1E-123 | Primer1E-123 | ATGGCCTGCTTGGTAAGCTA | GCGTGGGGAGAAGTTCAG |  | 56 | 116 | Ⅳ |
| TTE1E-124 | Primer1E-124 | GGGCCAGTAGGTCAACACAT | CCACGAGGTAGCAACAGAGA |  | 57 | 110 | Ⅳ |
| TTE1E-125 | Primer1E-125 | CTAAGAACAAGGCGGTTGTG | GGCATTCGTTTGTGTGAGTC | 1E | 55 | 145 | Ⅰ |
| TTE1E-126 | Primer1E-126 | GGTGGTAGTCCGTGATGGAT | TCAAAACTGGGCTCAGGA |  | 57 | 86 | Ⅴ |
| TTE1E-127 | Primer1E-127 | GCCACGCATAACCTGTGTAG | CCGTGGTGATGACAGAAATG | 1E | 56 | 165 | Ⅰ |
| TTE1E-128 | Primer1E-128 | GCTAGCCTTCAGCCCAATAA | TCCAGTTCGATTCTGGGAGT |  | 55 | 91 | Ⅳ |
| TTE1E-129 | Primer1E-129 | TTTCGGGCCATAGACCTG | GTCGAAACGGAGAAGAGTGG |  | 56 | 209 | Ⅳ |
| TTE1E-130 | Primer1E-130 | ACAACCTGACCCCACAACTC | CGCGCGGTAGTGATGATA | 1E, 2E | 56 | 112 | Ⅲ |
| TTE1E-131 | Primer1E-131 | CCTCTGTAGCCTCCAAATGC | CTAAGAACAAGGCGGTTGTG |  | 56 | 264 | Ⅳ |
| TTE1E-132 | Primer1E-132 | CCACACCACTTGTGTATCTGTG | GCAACAGAGACTTCCGATGA | 1E | 57 | 119 | Ⅰ |
| TTE1E-133 | Primer1E-133 | CCCTTAGGACAGGAGCTTCA | TTCGTTCTGCTCGTCTTCAC | 1E | 56 | 182 | Ⅰ |
| TTE1E-134 | Primer1E-134 | ACACCTTGTCAGCAGGGATT | CGAGCAAGCTACATCAAAGC |  | 55 | 109 | Ⅳ |
| TTE1E-135 | Primer1E-135 | GTTACTTTGGCTCGGGTTTG | CCCCTTAGGCTCATACTTCC | 1E | 56 | 151 | Ⅰ |
| TTE1E-136 | Primer1E-136 | CAGAATGGACAGTGCAGGAA | CTACCCGTCTCCTGAGCAAT | 1E | 56 | 107 | Ⅰ |
| TTE1E-137 | Primer1E-137 | AGGGTATTGACCGCATTGAC | GGCAAGGCAGTAGGAGGA | 1E | 56 | 107 | Ⅰ |
| TTE1E-138 | Primer1E-138 | GCCACTGACACACCTTACCA | TGCCAACTCGTACGCTACC |  | 57 | 110 | Ⅳ |
| TTE1E-139 | Primer1E-139 | TGGCCGAGTGTTTCTGAAG | GTCACACTCGGGAAACAACA | 1E | 55 | 121 | Ⅰ |
| TTE1E-140 | Primer1E-140 | GGTTCAGATGGTAACGACGAA | GTATGTGGGGTCGCCAAC | 1E | 56 | 113 | Ⅰ |
| TTE1E-141 | Primer1E-141 | GGAGGGAAGAAGGGCAATA | ACTGACTGCAAAAGGTGCTG | 1E | 55 | 141 | Ⅰ |
| TTE1E-142 | Primer1E-142 | GCGTTGGAGATGCTGAAGTA | CAGGATTGGGACCGTTAGAA | 1E | 55 | 101 | Ⅰ |
| TTE1E-143 | Primer1E-143 | GAGGTTAGGAGGGGATCTAGACA | GGTAACACCTTACTCCTGCTTG | 1E, 2E | 59 | 133 | Ⅲ |
| TTE1E-144 | Primer1E-144 | TAACCATCACGGGGAGTCA | CCTGCTAAGCCATCGTCTTC | 1E, 2E, 6E, 7E | 56 | 116 | Ⅲ |
| TTE1E-145 | Primer1E-145 | GGCTTGGGGTACGTTTTCT | TTACACAGGGCTTTGCAGTG | 1E | 55 | 102 | Ⅰ |
| TTE1E-146 | Primer1E-146 | CAAGGCAGTAGGCGGAAG | AGGGTATTGACCGCATTGAC | 1E | 56 | 105 | Ⅰ |
| TTE1E-147 | Primer1E-147 | GAGGCCCTCCTTGTCTACG | GGTCGTCAACATTCCAGTGA | 1E | 57 | 132 | Ⅰ |
| TTE1E-148 | Primer1E-148 | GAGCTGACATACCGACGATG | CAGTGCAGAAAAAGGCTAGG | 1E | 56 | 148 | Ⅰ |
| TTE1E-149 | Primer1E-149 | GCGTTGATCGTTGGATAGC | CGAAACAACCACCTACGACA |  | 54 | 103 | Ⅴ |
| TTE1E-150 | Primer1E-150 | CCACTCGGATGTGATCATTG | GCGACACAGAGGAACAAGTG | 1E | 56 | 108 | Ⅰ |
| TTE1E-151 | Primer1E-151 | ACGAGGAGGCTGACTGGTT | CTCTCTTCCACACCCATTCC | 1E | 57 | 119 | Ⅰ |
| TTE1E-152 | Primer1E-152 | AGGAGGGGAAGCTATGCAA | GTCAAGTTTGACCACGAGGA | 1E | 55 | 101 | Ⅰ |
| TTE1E-153 | Primer1E-153 | GGCTTACGGAAGTTTTCAGG | TCCAGACCCATTCCTCCTT | 1E | 55 | 101 | Ⅱ |
| TTE1E-154 | Primer1E-154 | CTCTCCCGGAGTACAAGTGC | TGGCATAGTGGACCGAAA | 1E | 56 | 103 | Ⅰ |
| TTE1E-155 | Primer1E-155 | CCCTTCGGGACTTTTGAA | TGGGTTTGGTTGTTGTCC |  | 54 | 101 | Ⅴ |
| TTE1E-156 | Primer1E-156 | TATGATGGGTCAGCAAGAGC | ACCTAGCTGTGGGAATCGTC | 1E | 56 | 102 | Ⅰ |
| TTE1E-157 | Primer1E-157 | ACAAGGTGATATGGCGTGTG | CAGTTTCCAGCCCTGTTCTC |  | 56 | 101 | Ⅳ |
| TTE1E-158 | Primer1E-158 | CAGCGATATGGTGGCACTT | TATCGCAGGAGCCATCAAG |  | 55 | 100 | Ⅳ |
| TTE1E-159 | Primer1E-159 | ACTAGGAGGGGGAGCTTCAG | AGTTCAAGCAGCCAAGGTTC |  | 57 | 130 | Ⅴ |
| TTE1E-160 | Primer1E-160 | TCAGTTCTCAGATCCCTCCA | CTGACTGCCACCAATTAGCA |  | 55 | 112 | Ⅴ |
| TTE1E-161 | Primer1E-161 | CTCGTCCACACCCATTCC | ACGAGGAGGCTGACTGGTT |  | 57 | 117 | Ⅴ |
| TTE1E-162 | Primer1E-162 | AGGACCTGGAATCAAACACG | GTACGCACCACTTTGCTTGA |  | 55 | 103 | Ⅴ |
| TTE1E-163 | Primer1E-163 | CAGTGAAGGGCGAAAGAAAG | CCCTTGCATCGAGAGAAAAG |  | 55 | 106 | Ⅴ |
| TTE1E-164 | Primer1E-164 | ACAGGTTATGCGTGGCTTC | GGTTGTGCTATGTCCGAAGA |  | 55 | 106 | Ⅳ |
| TTE1E-165 | Primer1E-165 | GTGTAACTCAAACCAGCAGTGG | CGGAGGGAGTAGATGGTACG |  | 59 | 114 | Ⅴ |
| TTE1E-166 | Primer1E-166 | GGAGGGAAGAAGGGCAATA | ACTGACTGCAAAAGGTGCTG |  | 55 | 121 | Ⅳ |
| TTE1E-167 | Primer1E-167 | GCCAAGCACAGTCACTGGTA | GGTGGAGACAAGGTAGGAAGG |  | 58 | 130 | Ⅳ |
| TTE1E-168 | Primer1E-168 | CCTGTCATTGGAGAAGTTGC | GCCTCCCAACATTGAGAACT |  | 55 | 105 | Ⅳ |
| TTE1E-169 | Primer1E-169 | GGTCGTCAACATTCCAGTGA | ATGCCCTCCTTGTCTACGTC |  | 54 | 131 | Ⅴ |
| TTE1E-170 | Primer1E-170 | AAACAGGGGGTGTCAACAGT | TGACCATCCTAGGTGGGTACTT |  | 56 | 116 | Ⅴ |
| TTE1E-171 | Primer1E-171 | TGATTCCCACGCATTGAG | ACCTGTCCAGTTGCGAGATT |  | 56 | 108 | Ⅳ |
| TTE1E-172 | Primer1E-172 | ACTGACTGCAAAAGGTGCTG | GGAGGGAAGAAGGGCAATA |  | 55 | 121 | Ⅳ |
| TTE1E-173 | Primer1E-173 | ATCGAGCATCTACGCATCCT | GCAACTTTGTCAGCTCCTCA |  | 57 | 113 | Ⅴ |
| TTE1E-174 | Primer1E-174 | ATGAAGCAACGACCCACTC | ACTTGACCTTTGGTGGTTGG |  | 55 | 126 | Ⅳ |
| TTE1E-175 | Primer1E-175 | GGTATGCACGCAGAAGTTCA | GAACACGGAAAGTCCGAAAC |  | 55 | 104 | Ⅴ |
| TTE1E-176 | Primer1E-176 | CGGTGCTGGTGTTTGTTG | CCAGGCTTCGACCACTATCT |  | 56 | 109 | Ⅴ |
| TTE1E-177 | Primer1E-177 | CTCTTCCACACCCATTCCAT | ACGAGGAGGCTGACTGGTT |  | 56 | 117 | Ⅴ |
| TTE1E-178 | Primer1E-178 | GGGTGTCACCAGTGCATTC | TGACCATCCTAGGTGGGTACTT |  | 58 | 109 | Ⅴ |
| TTE1E-179 | Primer1E-179 | GCCCTAGCTAGCAAAAGCAT | TGATGAACGAAGTGCTCACC |  | 55 | 126 | Ⅳ |
| TTE1E-180 | Primer1E-180 | CAGTTTCCAGCCCTGTTCTC | ACAAGGTGATATGGCGTGTG |  | 56 | 101 | Ⅴ |
| TTE1E-181 | Primer1E-181 | CTCAGGGTATTGACCGCATT | AAGGCAGTAGGAGGAAGCTG | 1E | 56 | 107 | Ⅰ |
| TTE1E-182 | Primer1E-182 | CTACCCGTCTCCTGAGCAAT | GAATCACGCAGAATGGACAG | 1E | 56 | 115 | Ⅰ |
| TTE1E-183 | Primer1E-183 | CAATGGCAAAACCCCAGT | GGAAGCCACGAGCTTATCC | 1E | 55 | 125 | Ⅰ |
| TTE1E-184 | Primer1E-184 | CAGATCACCAGCCTACAGGT | CCTTTCACCCAAGAGTCAAG | 1E | 56 | 112 | Ⅰ |
| TTE1E-185 | Primer1E-185 | ACGAGGAGGCTGACTGGTT | CTCTTCCACACCCATTCCAT | 1E | 56 | 117 | Ⅰ |
| TTE1E-186 | Primer1E-186 | CGCAAAATCTGGGATGAC | CCAGGCGGTGCAGTAAAT | 1E | 54 | 106 | Ⅱ |
| TTE1E-187 | Primer1E-187 | GGTTGTGCTATGTCCGAAGA | ACAGGTTATGCGTGGCTTC | 1E | 55 | 106 | Ⅰ |
| TTE1E-188 | Primer1E-188 | CCCAGACTTTGTCGAGCTTC | TCCTCATCGACGAGCTACC | 1E | 57 | 104 | Ⅰ |
| TTE1E-189 | Primer1E-189 | GCCAAGCACAGTCACTGGTA | GGTGGAGACAAGGTAGGAAGG | 1E | 58 | 130 | Ⅰ |
| TTE1E-190 | Primer1E-190 | ATGCCCTCCTTGTCTACGTC | GGTCGTCAACATTCCAGTGA | 1E | 56 | 131 | Ⅰ |
| TTE1E-191 | Primer1E-191 | CTTGTGTTGTGTTGCAGGTG | TTGCCTAGTATGGCGTGATG |  | 55 | 109 | Ⅳ |
| TTE1E-192 | Primer1E-192 | GCTGGGAGGCTAGCTACTTG | AGCAATGCTGAGAGGAGGAG | 1E | 58 | 113 | Ⅰ |
| TTE1E-193 | Primer1E-193 | CTCTTCCACACCCAGTCCAT | GGAGACGAGGAGGCTGACT | 1E | 58 | 121 | Ⅰ |
| TTE1E-194 | Primer1E-194 | GAACACGGAAAGTCCGAAAC | AAGGTATGCACGCAGAAGGT | 1E | 55 | 106 | Ⅰ |
| TTE1E-195 | Primer1E-195 | ACGAGGAGGCTGACTGGTT | CTCTTCCACACCCATTCCAT | 1E | 56 | 117 | Ⅰ |
| TTE1E-196 | Primer1E-196 | TGGCATAGTGGACCGAAA | CTCTCCCGGAGTACAAGTGC | 1E | 56 | 103 | Ⅰ |
| TTE1E-197 | Primer1E-197 | TGACCATCCTAGGTGGGTACTT | AAACAGGGGGTGTCAACAGT | 1E | 57 | 116 | Ⅰ |
| TTE1E-198 | Primer1E-198 | ACCTAGCTGTGGGAATCGTC | TATGATGGGTCAGCAAGAGC | 1E | 56 | 102 | Ⅰ |
| TTE1E-199 | Primer1E-199 | CACCAGCCTACAGGTACCAA | GTAGCACGGGTGCTCAAAA | 1E | 56 | 127 | Ⅰ |
| TTE1E-200 | Primer1E-200 | CTTGTGTTGTGTTGCAGGTG | TTGCCTAGTATGGCGTGATG |  | 55 | 109 | Ⅳ |
| TTE1E-201 | Primer1E-201 | GAACACGGAAAGTCCGAAAC | AAGGTATGCACGCAGAAGGT | 1E | 55 | 106 | Ⅰ |
| TTE1E-202 | Primer1E-202 | CCTGCTAAGCCATCGTCTTC | TAACCATCACGGGGAGTCA | 1E | 56 | 116 | Ⅰ |
| TTE1E-203 | Primer1E-203 | ATCGGAGATGGGATAACGTG | AAACAGTCACCATCGACCAC |  | 55 | 119 | Ⅳ |
| TTE1E-204 | Primer1E-204 | CTCTCCCGGAGTACAAGTGC | TGGCATAGTGGACCGAAA | 1E | 56 | 103 | Ⅰ |
| TTE1E-205 | Primer1E-205 | TCGGGGTCTAGTCTGAACAA | TTCAGTGCGTAGCGGAGTAA | 1E | 55 | 103 | Ⅱ |
| TTE1E-206 | Primer1E-206 | CTGTCTTGAGTAGGGGCAAAG | CAGATCAAGAGGGACCTACACA | 1E | 58 | 106 | Ⅰ |
| TTE1E-207 | Primer1E-207 | AAGGTATGCACGCAGAAGGT | GAACACGGAAAGTCCGAAAC | 1E | 55 | 106 | Ⅰ |
| TTE1E-208 | Primer1E-208 | ACTGACTGCAAATGGTGCTG | GGAGGGAAGAAGGGCAATA | 1E | 55 | 121 | Ⅰ |
| TTE1E-209 | Primer1E-209 | CTCTTCCACACCCATTCCAT | AGGCTGACTGGTGTGGTTTT | 1E | 55 | 111 | Ⅰ |
| TTE1E-210 | Primer1E-210 | CCCTAGCCAGGACAGACATT | TGTCGCTGTATTGCACACTG | 1E | 56 | 118 | Ⅰ |
| TTE1E-211 | Primer1E-211 | GGCGACAAAGAGGAACAAGT | CCACTCGGATGTGATCATTG | 1E | 55 | 109 | Ⅰ |
| TTE1E-212 | Primer1E-212 | ATCGAGCATCTACGCATCCT | GCAACTTTGTCAGCTCCTCA | 1E | 55 | 113 | Ⅱ |
| TTE1E-213 | Primer1E-213 | AACAACCCGAGTGGTGTCA | TGTGAGTTCTTCAGCGCAGT |  | 55 | 138 | Ⅳ |
| TTE1E-214 | Primer1E-214 | TGGAGCTCCTCCACATTCTT | GGTCCAAGTGTGCTTTTCCT | 1E | 55 | 135 | Ⅱ |
| TTE1E-215 | Primer1E-215 | AGCTTCCTCTGTATCCACCAG | AGTCTTTCGATCAGGACACG | 1E | 57 | 102 | Ⅰ |
| TTE1E-216 | Primer1E-216 | TGGCATAGTGGACCGAAA | CTCTCCCGGAGTACAAGTGC | 1E | 56 | 103 | Ⅰ |
| TTE1E-217 | Primer1E-217 | CTCTTCCACACCCATTCCAT | ACGAGGAGGCTGACTGGTT | 1E | 56 | 117 | Ⅰ |
| TTE1E-218 | Primer1E-218 | TGAGCTTGTCAGCCAGGTAG | CCTCGATGACAGGATGAAGA | 1E | 56 | 116 | Ⅰ |
| TTE1E-219 | Primer1E-219 | GAACACGGAAAGTCCGAAAC | AAGGTATGCACGCAGAAGGT | 1E | 55 | 106 | Ⅰ |
| TTE1E-220 | Primer1E-220 | TGTGGGCACAGTCCATACTC | ACCACACTGGCTCTTTGCTT | 1E | 56 | 106 | Ⅰ |
| TTE1E-221 | Primer1E-221 | ATGCCCTCCTTGTCTACGTC | GGTCGTCAACATTCCAGTGA | 1E | 56 | 131 | Ⅰ |
| TTE1E-222 | Primer1E-222 | CCACAAGCTTTAGAGACCTCCT | TGTAGTCTTAGGCTCCTCCTTACC | 1E | 59 | 107 | Ⅱ |
| TTE1E-223 | Primer1E-223 | GACCATTACCTGGAGGATGC | GGGCTGTCATTTGAAGTGTG | 1E | 56 | 121 | Ⅰ |
| TTE1E-224 | Primer1E-224 | ACGAGTCGGGTGGTGTATTC | CAACGGAACGCTAGGTCAA | 1E | 56 | 110 | Ⅱ |
| TTE1E-225 | Primer1E-225 | ACGAGTCGGGTGGTGTATTC | CAACGGAACGCTAGGTCAA | 1E | 56 | 110 | Ⅱ |
| TTE1E-226 | Primer1E-226 | GCCAAGCACAGTCACTGGTA | GGTGGAGACAAGGTAGGAAGG | 1E | 58 | 130 | Ⅰ |
| TTE1E-227 | Primer1E-227 | CCACACATAGCCCATCTTGA | CATACCACGCGGAGGTAGA |  | 56 | 108 | Ⅴ |
| TTE1E-228 | Primer1E-228 | TGATGAACGAAGTGCTCACC | GCCCTAGCTAGCAAAAGCAT | 1E | 55 | 126 | Ⅰ |
| TTE1E-229 | Primer1E-229 | TGCGAGATTGCTCTGCTC | GCGTGTGGCTGAAGTACAAA | 1E | 55 | 107 | Ⅰ |
| TTE1E-230 | Primer1E-230 | TGGCTTCGTGGTTTCTACCT | GCCTCGATCTCCTCCAAAG | 1E | 56 | 102 | Ⅰ |
| TTE1E-231 | Primer1E-231 | ATGCCCTCCTTGTCTACGTC | GGTCGTCAACATTCCAGTGA | 1E | 56 | 131 | Ⅰ |
| TTE1E-232 | Primer1E-232 | GGGAGAAGAGGAGGCTGAAT | TCTCTTCCCCACCAATTCC |  | 56 | 123 | Ⅴ |
| TTE1E-233 | Primer1E-233 | ATGAAGCAACGACCCACTC | ACTTGACCTTTGGTGGTTGG |  | 55 | 126 | Ⅳ |
| TTE1E-234 | Primer1E-234 | AGTCTTTCGATCAGGACACG | AGCTTCCTCTGTATCCACCAG | 1E | 57 | 102 | Ⅰ |
| TTE1E-235 | Primer1E-235 | TGACCATCCTAGGTGGGTACTT | ACAACAGTGCATTCGACCTG | 1E | 57 | 104 | Ⅰ |

Note: Types I: amplified specific bands only from PI531750, 8801 and TDS1E/1D

Types II: amplified specific bands only from PI531718, PI531750, 8801 and TDS1E/1D

Types III: amplified specific bands from PI531750, 8801, TDS1E/1D, and PI531718 or other substitution lines

Types IV: no specific bands

Types V: no any amplicon
