# Supplementary material for: Characterization of a wheat–tetraploid Thinopyrum elongatum 1E(1D) substitution line K17–841-1 by cytological and phenotypic analysis and developed molecular markers
Source: BMC Genomics. 2019 Dec 10;20:963. doi: 10.1186/s12864-019-6359-9 (PMC6905003; doi:10.1186/s12864-019-6359-9)
Supplement: Supplementary file 3 — Additional file 3: Table S3. Stripe rust response at seedling stage and specific amplification of 1E chromosome markers in F2 population. [file 12864_2019_6359_MOESM3_ESM.docx]

**Table S3 Stripe rust response at seedling stage and specific amplification of 1E chromosome markers in F_2_ population**

| Lines | Infection type | Resistance/susceptibility | Specific amplicon |
| --- | --- | --- | --- |
| SY95-71 | 4 | S |  |
| SM482 | 4 | S | 0 |
| SM921 | 4 | S | 0 |
| SM969 | 4 | S | 0 |
| 8801 | 0; | R | 1 |
| K17-841-1 | 0 | R | 1 |
| F_2_-1 | 4 | S | 0 |
| F_2_-2 | 4 | S | 0 |
| F_2_-3 | 0 | R | 1 |
| F_2_-4 | 4 | S | 0 |
| F_2_-5 | 4 | S | 0 |
| F_2_-6 | 0 | R | 1 |
| F_2_-7 | 4 | S | 0 |
| F_2_-8 | 4 | S | 0 |
| F_2_-9 | 4 | S | 0 |
| F_2_-10 | 4 | S | 0 |
| F_2_-11 | 4 | S | 0 |
| F_2_-12 | 4 | S | 0 |
| F_2_-13 | 0 | R | 1 |
| F_2_-14 | 4 | S | 0 |
| F_2_-15 | 4 | S | 0 |
| F_2_-16 | 4 | S | 0 |
| F_2_-17 | 4 | S | 0 |
| F_2_-18 | 4 | S | 0 |
| F_2_-19 | 4 | S | 0 |
| F_2_-20 | 4 | S | 0 |
| F_2_-21 | 0 | R | 1 |
| F_2_-22 | 0 | R | 1 |
| F_2_-23 | 4 | S | 0 |
| F_2_-24 | 0 | R | 1 |
| F_2_-25 | 4 | S | 0 |
| F_2_-26 | 4 | S | 0 |
| F_2_-27 | 4 | S | 0 |
| F_2_-28 | 4 | S | 0 |
| F_2_-29 | 4 | S | 0 |
| F_2_-30 | 0 | R | 1 |
| F_2_-31 | 4 | S | 0 |
| F_2_-32 | 0 | R | 1 |
| F_2_-33 | 4 | S | 0 |
| F_2_-34 | 4 | S | 0 |
| F_2_-35 | 4 | S | 0 |
| F_2_-36 | 0 | R | 1 |
| F_2_-37 | 0 | R | 1 |
| F_2_-38 | 4 | S | 0 |
| F_2_-39 | 4 | S | 0 |
| F_2_-40 | 4 | S | 0 |
| F_2_-41 | 4 | S | 0 |
| F_2_-42 | 0 | R | 1 |
| F_2_-43 | 4 | S | 0 |
| F_2_-44 | 4 | S | 0 |
| F_2_-45 | 4 | S | 0 |
| F_2_-46 | 4 | S | 0 |
| F_2_-47 | 0 | R | 1 |
| F_2_-48 | 0 | R | 1 |
| F_2_-49 | 4 | S | 0 |
| F_2_-50 | 4 | S | 0 |
| F_2_-51 | 4 | S | 0 |
| F_2_-52 | 4 | S | 0 |
| F_2_-53 | 4 | S | 0 |
| F_2_-54 | 4 | S | 0 |
| F_2_-55 | 0 | R | 1 |
| F_2_-56 | 0 | R | 1 |
| F_2_-57 | 4 | S | 0 |
| F_2_-58 | 4 | S | 0 |
| F_2_-59 | 4 | S | 0 |
| F_2_-60 | 4 | S | 0 |
| F_2_-61 | 0 | R | 1 |
| F_2_-62 | 4 | S | 0 |
| F_2_-63 | 4 | S | 0 |
| F_2_-64 | 0 | R | 1 |
| F_2_-65 | 4 | S | 0 |
| F_2_-66 | 4 | S | 0 |
| F_2_-67 | 0 | R | 1 |
| F_2_-68 | 4 | S | 0 |
| F_2_-69 | 4 | S | 0 |
| F_2_-70 | 4 | S | 0 |
| F_2_-71 | 4 | S | 0 |
| F_2_-72 | 4 | S | 0 |
| F_2_-73 | 0 | R | 1 |
| F_2_-74 | 4 | S | 0 |
| F_2_-75 | 0 | R | 1 |
| F_2_-76 | 4 | S | 0 |
| F_2_-77 | 4 | S | 0 |
| F_2_-78 | 4 | S | 0 |
| F_2_-79 | 4 | S | 0 |
| F_2_-80 | 4 | S | 0 |

Note: “1” or “0” indicates the presence or absence of the specific band, respectively.
